# Sodium Phenylbutyrate Controls Neuroinflammatory and Antioxidant Activities and Protects Dopaminergic Neurons in Mouse Models of Parkinson’s Disease

**DOI:** 10.1371/journal.pone.0038113

**Published:** 2012-06-18

**Authors:** Avik Roy, Anamitra Ghosh, Arundhati Jana, Xiaojuan Liu, Saurav Brahmachari, Howard E. Gendelman, Kalipada Pahan

**Affiliations:** 1 Department of Neurological Sciences, Rush University Medical Center, Chicago, Illinois, United States of America; 2 Section of Neuroscience, University of Nebraska Medical Center College of Dentistry, Lincoln, Nebraska, United States of America; 3 Department of Pharmacology and Experimental Neuroscience, University of Nebraska Medical Center, Omaha, Nebraska, United States of America; Emory University, United States of America

## Abstract

Neuroinflammation and oxidative stress underlie the pathogenesis of various neurodegenerative disorders. Here we demonstrate that sodium phenylbutyrate (NaPB), an FDA-approved therapy for reducing plasma ammonia and glutamine in urea cycle disorders, can suppress both proinflammatory molecules and reactive oxygen species (ROS) in activated glial cells. Interestingly, NaPB also decreased the level of cholesterol but involved only intermediates, not the end product of cholesterol biosynthesis pathway for these functions. While inhibitors of both geranylgeranyl transferase (GGTI) and farnesyl transferase (FTI) inhibited the activation of NF-κB, inhibitor of GGTI, but not FTI, suppressed the production of ROS. Accordingly, a dominant-negative mutant of p21^rac^, but not p21^ras^, attenuated the production of ROS from activated microglia. Inhibition of both p21^ras^ and p21^rac^ activation by NaPB in microglial cells suggests that NaPB exerts anti-inflammatory and antioxidative effects via inhibition of these small G proteins. Consistently, we found activation of both p21^ras^ and p21^rac^ *in vivo* in the substantia nigra of acute 1-methyl-4-phenyl-1,2,3,6-tetrahydropyridine (MPTP) mouse model of Parkinson’s disease. Oral administration of NaPB reduced nigral activation of p21^ras^ and p21^rac^, protected nigral reduced glutathione, attenuated nigral activation of NF-κB, inhibited nigral expression of proinflammatory molecules, and suppressed nigral activation of glial cells. These findings paralleled dopaminergic neuronal protection, normalized striatal neurotransmitters, and improved motor functions in MPTP-intoxicated mice. Consistently, FTI and GGTI also protected nigrostriata in MPTP-intoxicated mice. Furthermore, NaPB also halted the disease progression in a chronic MPTP mouse model. These results identify novel mode of action of NaPB and suggest that NaPB may be of therapeutic benefit for neurodegenerative disorders.

## Introduction

Neurodegenerative disorders are a group of devastating disorders of the central nervous system, in which progressive loss of structure and function of neurons, including neuronal death is observed. With the progress of research, many similarities are appearing that ultimately relate these diseases to one another on a subcellular level. For example, recent studies demonstrate that neuroinflammation and oxidative stress are two hallmarks of various neurodegenerative disorders [1], [2]. In neurodegenerative disorders, microglial activation is in close proximity to damaged or dying dopaminergic neurons. Among various mediators capable of promoting neurodegeneration are microglial-derived reactive oxygen species (ROS) and a significant source of ROS under such pathological conditions is NADPH-oxidase, which is a multimeric enzyme composed of gp91^phox^, p22^phox^, p47^phox^, p67^phox^, and p40^phox^ subunits [3]. It has been demonstrated that either gp91^phox^ inactivation [4] or a chemical inhibitor of NADPH oxidase [5] protects neurons in various models of neurodegenerative disorders. In addition to ROS, the concentration of NO_2_^−^ (nitrite), a metabolite of nitric oxide (NO), increases in the CSF of patients with Parkinson’s disease and Alzheimer’s disease in comparison age-matched controls. Similarly, a variety of pro-inflammatory cytokines including tumor necrosis factor alpha (TNF-α), interleukin-1beta (IL-1β), IL-6, eicosanoids, and other immune neurotoxins are found in either CSF or affected brain regions in neurodegenerative disorders [6]. Therefore, inflammation and oxidative stress are important targets for neuronal protection in neurodegenerative disorders.

Sodium phenylbutyrate (NaPB) is a FDA-approved drug against urea cycle disorders in human. Here we delineate new beneficial properties of NaPB. Interestingly, NaPB suppressed the activation of NF-κB, inhibited the expression of proinflammatory molecules and attenuated the production of ROS from activated microglia via modulation of the mevalonate pathway and associated activation of small G proteins like p21^ras^ and p21^rac^. We also demonstrate that oral administration of NaPB inhibited nigral activation of p21^ras^ and p21^rac^, suppressed nigral expression of proinflammatory molecules, improved the level of master antioxidant GSH, and exhibited significant protection of the nigrostriatal axis after MPTP intoxication. These results provide new modes of action of NaPB and open an option for treating patients with neurodegenerative disorders with this FDA-approved drug as primary or adjunct therapy.

## Materials and Methods

Animal maintaining and experiments were in accordance with National Institute of Health guidelines and were approved by the Institutional Animal Care and Use committee (IACUC#06-048) of the Rush University of Medical Center, Chicago, IL.

### Isolation of Mouse Microglia

Microglia were isolated from mixed glial cultures according to the procedure of Giulian and Baker [7] with modifications as previously described [8], [9]. Briefly, on day 7–9, the mixed glial cultures were washed three times with Dulbecco’s modified Eagle’s medium/F-12 and subjected to shaking at 240 rpm for 2 h at 37°C on a rotary shaker. The floating cells were washed and seeded on to plastic tissue culture flasks and incubated at 37°C for 1 h. The attached cells were seeded onto new plates for further studies. Ninety to ninety-five percent of cells were found to be positive for Mac-1. Mouse BV-2 microglial cells (kind gift from Virginia Bocchini of University of Perugia) were also maintained and induced as indicated above.

### Isolation of Primary Human Astroglia

Primary human astroglia were prepared as previously described [10], [11], [12]. Fetal brain tissues were obtained from the Human Embryology Laboratory (University of Washington, Seattle, WA, USA). Briefly, 11- to 17-week-old fetal brains were dissociated by trituration and trypsinization. On 9^th^ day, these mixed glial cultures were placed on a rotary shaker at 240 rpm at 37°C for 2 h to remove loosely attached microglia. Then on 11^th^ day, flasks were shaken again at 190 rpm at 37°C for 18 h to remove oligodendroglia. The attached cells remaining were primarily astrocytes. These cells were trypsinized and subcultured to yield more viable and healthy cells. More than 98% of these cells obtained by this method were positive for glial fibrillary acidic protein (GFAP), an astrocyte marker.

### Immunoblot Analysis

Immunoblot analysis was carried out as described earlier [13], [14]. Briefly, cells homogenates were electrophoresed, proteins were transferred onto a nitrocellulose membrane, and protein band was visualized with Odyssey infrared scanner after immunolabeling with primary antibodies followed by infra-red fluorophore-tagged secondary antibody (Invitrogen, Carlsbad, CA).

### Electrophoretic Mobility Shift Assay (EMSA)

Nuclear extracts were prepared and EMSA was carried out as previously described [9], [10], [15]. Briefly, IRDye™ infrared dye end-labeled oligonucleotides containing the consensus binding sequence for NF-κB (5′-AGT TGA GGG GAC TTT CCC AGG C-3′) were purchased from LI-COR Biosciences (Lincoln, NE). Six-micrograms of nuclear extract was incubated with binding buffer and with IR-labeled probe for 20 min. Subsequently, samples were separated on a 6% polyacrylamide gel in 0.25x TBE buffer (Tris borate-EDTA) and analyzed by Odyssey Infrared Imaging System (LI-COR Biosciences).

### Transcriptional Activity of NF-κB

Transcriptional activity of NF-κB was assayed as previously described [9], [10], [15]. Briefly, cells plated at 50 to 60% confluence in 12-well plates were transfected with 0.25 µg pBIIX-Luc (an NF-κB-dependent reporter construct) and 12.5 ng pRL-TK (a plasmid encoding Renilla luciferase, used as transfection efficiency control; Promega, Madison, WI) using LipofectAMINE Plus (Invitrogen, Carlsbad, CA). After 24 h of transfection, cells were stimulated with MPP^+^ for an additional 6 h, and firefly and Renilla luciferase activities were recorded in a TD-20/20 Luminometer (Turner Designs, Sunnyvale, CA) by analyzing total cell extract according to standard instructions provided in the Dual Luciferase Kit (Promega). Relative luciferase activity of cell extracts was typically represented as (firefly luciferase value/Renilla luciferase value) × 10^−3^.

### Chromatin Immunoprecipitation (ChIP)

ChIP assays were performed as described earlier [9] using a ChIP assay kit (Upstate Biotechnology, Lake Placid, NY). Briefly, 2×10^6^ microglial cells preincubated with gemfibrozil for 6 h were stimulated with LPS. After 3 h of stimulation, cells were fixed by adding formaldehyde (1% final concentration), and cross-linked adducts were resuspended and sonicated, resulting in an average chromatin fragment size of 400 bp. ChIP was performed on the cell lysate by overnight incubation at 4°C with 2 µg of antibodies against p65 followed by incubation with protein G agarose (Santa Cruz Biotechnology) for 2 h. The beads were washed and incubated with elution buffer. To reverse the cross-linking and purify the DNA, precipitates were incubated in a 65°C incubator overnight and digested with proteinase K. DNA samples were then purified, precipitated, and precipitates were washed with 75% ethanol, air-dried, and resuspended in TE buffer. Following primers were used to amplify fragments flanking proximal NF-κB elements in the mouse iNOS promoter:

Sense: 5′-CAT GAG GAT ACA CCA CAG AG-3′.

Antisense: 5′-AAG ACC CAA GCG TGA GGA GC-3′.

Following primers were used to amplify fragments flanking distal NF-κB elements in the mouse iNOS promoter:

Sense: TGC TAG GGG GAT TTT CCC TCT CTC-3′.

Antisense: 5′-ACC CTG TTC TGA GAA ACA AA-3′.

Sense: 5′-GAT GTG CTA GGG GGA TTT TCC C-3′.

Antisense: 5′-TGG GCT AGC CTG GTC TAC AGA G-3′.

The PCRs were repeated by using varying cycle numbers and different amounts of templates in order to ensure that results were in the linear range of PCR.

### Monitoring Microglial ROS Production

Cells, cultured in 8-well chamber slides, were treated with MPP^+^ under serum-free condition. At different time points of stimulation, supernatants were removed and cells were washed with Hank’s buffered salt solution (HBSS) followed by addition of 100 µl of 25 µM carboxy-H_2_DCFDA to each well for 30 min of incubation as described by us [16]. During the last five minutes of incubation, Hoechst 33342 was added to each well at a dilution of 1∶1000 for staining nuclei. Cells were then washed with HBSS, mounted with DPX mounting media and observed under an Olympus IX81 fluorescent microscope.

### Superoxide Measurements

Superoxide production was detected by LumiMax™ Superoxide Anion Detection Kit (Stratagene) as described by us [30].

### Assay of Cholesterol in Serum

Total cholesterol was quantified in serum by using an Amplex Red Cholesterol Assay kit from Invitrogen. Briefly, cholesterol was oxidized by cholesterol oxidase to yield H_2_O_2_, which then reacted with 10-acetyl-3,7 dihydroxyphenoxazine (Amplex Red). In the presence of horseradish peroxidase (HRP), this Amplex Red:H_2_O_2_ complex produced highly fluorescent resorufin, which was detected by fluorometry.

### Animals and MPTP Intoxications

Six- to eight-week old C57BL/6 mice were purchased from Harlan, Indianapolis, IN. For acute MPTP intoxication, mice received four intraperitoneal (i.p.) injections of MPTP-HCl (18 mg/kg of free base; Sigma Chemical Co., St. Louis, MO) in saline at 2 hr intervals [9], [17]. Control animals received only saline. For chronic MPTP intoxication, mice received 10 injections of MPTP (s.c.; 25 mg/kg body weight) together with 10 injections of probenecid (i.p.; 250 mg/kg body weight) at an interval of 3.5 d [18], [19].

### Drugs and Antibodies

NaPB, farnesyl transferase inhibitor (FTI), geranylgeranyl transferase inhibitor (GGTI), and rabbit anti-mouse iNOS were obtained from Calbiochem, Gibbstown, NJ. Rabbit and goat anti-NF-κB p65 and goat anti-glial fibrillary acidic protein (GFAP) were purchased from Santa Cruz Biotechnology (Santa Cruz, CA). Rat anti-mouse CD11b and mouse anti-human CD11b were purchased from Abcam (Cambridge, MA) and Serotec (Raleigh, NC), respectively. Cy2- and Cy5-conjugated antibodies were obtained from Jackson Immuno Research Laboratories (West Grove, PA).

### Drug Treatments

NaPB is a FDA-approved drug for patients with urea cycle disorders and its recommended dose for affected children is 400–600 mg/kg/day. However, because either PD patients or MPTP-intoxicated mice do not suffer from urea cycle disorders, we have reduced the dose for treating mice. Therefore, for short-term treatment, mice with acute MPTP intoxication received NaPB (200 mg/kg body wt/d) in a volume of 100 µl water via gavage from 3 h after the last injection of MPTP. The neurotoxic effect of MPTP depends on several key toxicokinetic steps such as its conversion into MPP^+^ in glial cells by MAO-B and the uptake of MPP^+^ by dopaminergic neurons. Przedborski and colleagues [20] have shown that sufficient amount of MPTP is converted into MPP^+^ within 90 min of the last injection of MPTP in an acute MPTP model. Therefore, to avoid any possible influence of NaPB on entry and conversion of MPTP into MPP^+^ in the midbrain, oral treatment began 3 h after the last injection of MPTP. On the other hand, for long-term treatment, mice with chronic MPTP intoxication received a lower dose of NaPB (100 mg/kg body wt/d) via gavage from the 3^rd^ injection of MPTP/probenecid. Control MPTP mice received only 100 µl water via gavage everyday.

On the other hand, FTI and GGTI were solubilized in normal saline and mice were treated with FTI and GGTI daily via intraperitoneal (i.p.) injection at doses of 5 or 10 mg/kg body wt/d starting from 3 h after the last injection of MPTP.

### Activation of p21^ras^ and p21^rac^

Activation of p21^ras^ was monitored as described before [17], [21]. Briefly, after 6 h of MPTP insult, ventral midbrain was dissected out and frozen immediately on dry ice. The p21^ras^-binding domain (RBD) of the p21^ras^ effector kinase Raf1 has been shown to bind specifically to the GTP-bound (active) form of p21^ras^ proteins. Therefore, using an assay kit from Upstate Biotechnology (Waltham, MA), ventral midbrain tissues were homogenized with lysis buffer containing inhibitors of different proteases and kinases followed by immuno-pull down of active p21^ras^ using Raf-RBD-GST beads. Then the amount of activated p21^ras^ was determined in GST beads by a Western blot using a p21^ras^ specific antibody.

As activated p21^ras^ interacts with Raf1, activated p21^rac^ interacts with p21-activated kinase (PAK). Accordingly, p21^rac^-interacting domain (RID) of PAK binds specifically to the GTP-bound (active) form of p21^rac^. Therefore, using an assay kit from Upstate Biotechnology (Waltham, MA), PAK-RID-GST beads were used to immuno-pull down active p21^rac^ from cell lysates followed by a Western blot using a p21^rac^ specific antibody.

### Semi-quantitative RT-PCR Analysis for Proinflammatory Molecules (iNOS, IL-1β and TNF-α) and Glial Cell Markers

Total RNA was isolated from ventral midbrain using Ultraspec-II RNA reagent (Biotecx Laboratories, Inc., Houston, TX) following manufacturer’s protocol. To remove any contaminating genomic DNA, total RNA was digested with DNase. RT-PCR was carried out as described earlier [9], [15], [17] using a RT-PCR kit (Clontech, Mountain View, CA) and following primers.

iNOS: Sense: 5′-CCCTTCCGAAGTTTCTGGCAGCAGC-3′.

Antisense: 5′-GGCTGTCAGAGCCTCGTGGCTTTGG-3′.

IL-1β: Sense: 5′-CTCCATGAGCTTTGTACAAGG-3′.

Antisense: 5′-TGCTGATGTACCAGTTGGGG-3′.

TNF-α: Sense: 5′-TTCTGTCTACTGAACTTCGGGGTGATCGGTCC-3′.

Antisense: 5′-GTATGAGATAGCAAATCGGCTGACGGTGTGGG-3′.

CD11b: Sense: 5′-GTGAGGATTCCTACGGGACCCAGGT-3′.

Antisense: 5′-GGCGTACTTCACAGGCAGCTCCAAC-3′.

GFAP: Sense: 5′- GGCGCTCAATGCTGGCTTCA-3′.

Antisense: 5′- TCTGCCTCCAGCCTCAGGTT-3′.

GAPDH: Sense: 5′-GGTGAAGGTCGGTGTGAACG-3′.

Antisense: 5′-TTGGCTCCACCCTTCAAGTG-3′.

### Real-time PCR Analysis

It was performed in the ABI-Prism7700 sequence detection system (Applied Biosystems, Foster City, CA) as described earlier [9], [15], [17] using TaqMan Universal Master mix and optimized concentrations of FAM-labeled probes and primers. Data were processed by the ABI Sequence Detection System 1.6 software.

### Immunohistochemistry and Quantitative Morphology

Seven days after MPTP intoxication, mice were sacrificed and their brains fixed, embedded, and processed for tyrosine hydroxylase (TH) staining as previously described [9], [17], [22]. Total numbers of TH-positive neurons in SNpc were counted stereologically with STEREO INVESTIGATOR software (MicroBrightfield, Williston, VT) by using an optical fractionator. Quantitation of striatal TH immunostaining was performed as described [9], [17], [22]. Optical density measurements were obtained by digital image analysis (Scion, Frederick, MD). Striatal TH optical density reflected dopaminergic fiber innervation. For immunofluorescence staining on fresh frozen sections, rat anti-mouse CD11b (1∶100), goat anti-mouse GFAP (1∶100), rabbit anti NF-κB p65 (1∶100), goat anti-NF-κB p65 (1∶100), rabbit anti NF-κB p50 (1∶100), and rabbit anti-mouse iNOS (1∶250) were used. The samples were mounted and observed under a Bio-Rad MRC1024ES confocal laser scanning microscope.

### HPLC Analysis of Striatal Dopamine and its Metabolite Levels

Striatal level of dopamine, DOPAC (3, 4-dihydroxyphenylacetic acid) and HVA (homovanillic acid) was quantified as described earlier [9], [17], [19]. Briefly, mice were sacrificed by cervical dislocation after 7 days of MPTP intoxication and their striata were collected and immediately frozen in dry ice and stored at −80°C until analysis. On the day of the analysis, tissues were sonicated in 0.2 M perchloric acid containing isoproterenol and resulting homogenates were centrifuged at 20,000 × g for 15 min at 4C. After pH adjustment and filtration, 10 µl of supernatant was injected onto an Eicompak SC-3ODS column (Complete Stand-Alone HPLC-ECD System EiCOMHTEC-500 from JM Science Inc., Grand Island, NY) and analyzed following manufacturer’s protocol.

### Analysis of GSH

Nigral tissues were dissected and then sonicated in 0.2 M perchloric acid solution followed by centrifugation of nigral extracts at 12,000 rpm for 10 mins at 4°C. Resulting supernatants were analyzed for GSH in Complete Stand-Alone HPLC-ECD System EiCOMHTEC-500 using gold working electrode (Eicom We-AU) and mobile phase containing 99% 0.1 M sodium phosphate buffer (pH 2.5), 1% MeOH (v/v), and 50 mg/L EDTA-2Na.

### Behavioral Analyses

Two types of behavioral experiments were conducted. This included open field experiment for locomotor activity and rotorod experiment for feet movement as described earlier [9], [17], [19]. Locomotor activity was measured after 7 d of the last dose of MPTP injection in Digiscan Monitor (Omnitech Electronics, Inc., Columbus, OH). This Digiscan Monitor records stereotypy and rearing, behaviors that are directly controlled by striatum, as well as other basic locomotion parameters, such as horizontal activity, total distance traveled, number of movements, movement time, rest time, mean distance, mean time, center time etc. Before any insult or treatment, mice were placed inside the Digiscan Infra-red Activity Monitor for 10 min daily and on rotorod for 10 min daily for 3 consecutive days to train them and record their baseline values. Briefly, animals were removed directly from their cages and gently placed nose first into a specified corner of the open-field apparatus and after release, data acquisition began at every 5 min interval. DIGISCAN software was used to analyze and store horizontal and vertical activity data, which were monitored automatically by infra-red beams. In rotorod, the feet movement of the mice was observed at different speeds. To eliminate stress and fatigues, mice were given a 5-min rest interval. Then 7 d after the last dose of MPTP, open field and rotorod tests were carried out twice at 6 h interval on each mouse separately [9], [17]. Locomotor activity measures were assessed after baseline value comparison.

### Statistics

All values are expressed as means ± SEM. Differences among means were analyzed by one- or two-way ANOVA considering time, dose or treatment as the independent factor. The one way ANOVA was performed while analyzing dose-dependent effect of NaPB on the induction of NO production or the activation of NF-κB in activated microglial cells. On the other hand, two-way ANOVA was employed to analyze the effect of Δp21^rac^ or Δp21^ras^ on LPS-induced time-dependent production of superoxide. In other cases, Student’s *t*-test was used to compare outcome between two groups (e.g. control vs MPTP, MPTP vs NaPB etc).

## Results

### NaPB Attenuates the Expression of iNOS and Proinflammatory Cytokines in Activated Mouse Microglia and Human Astroglia

**Figure 1:**
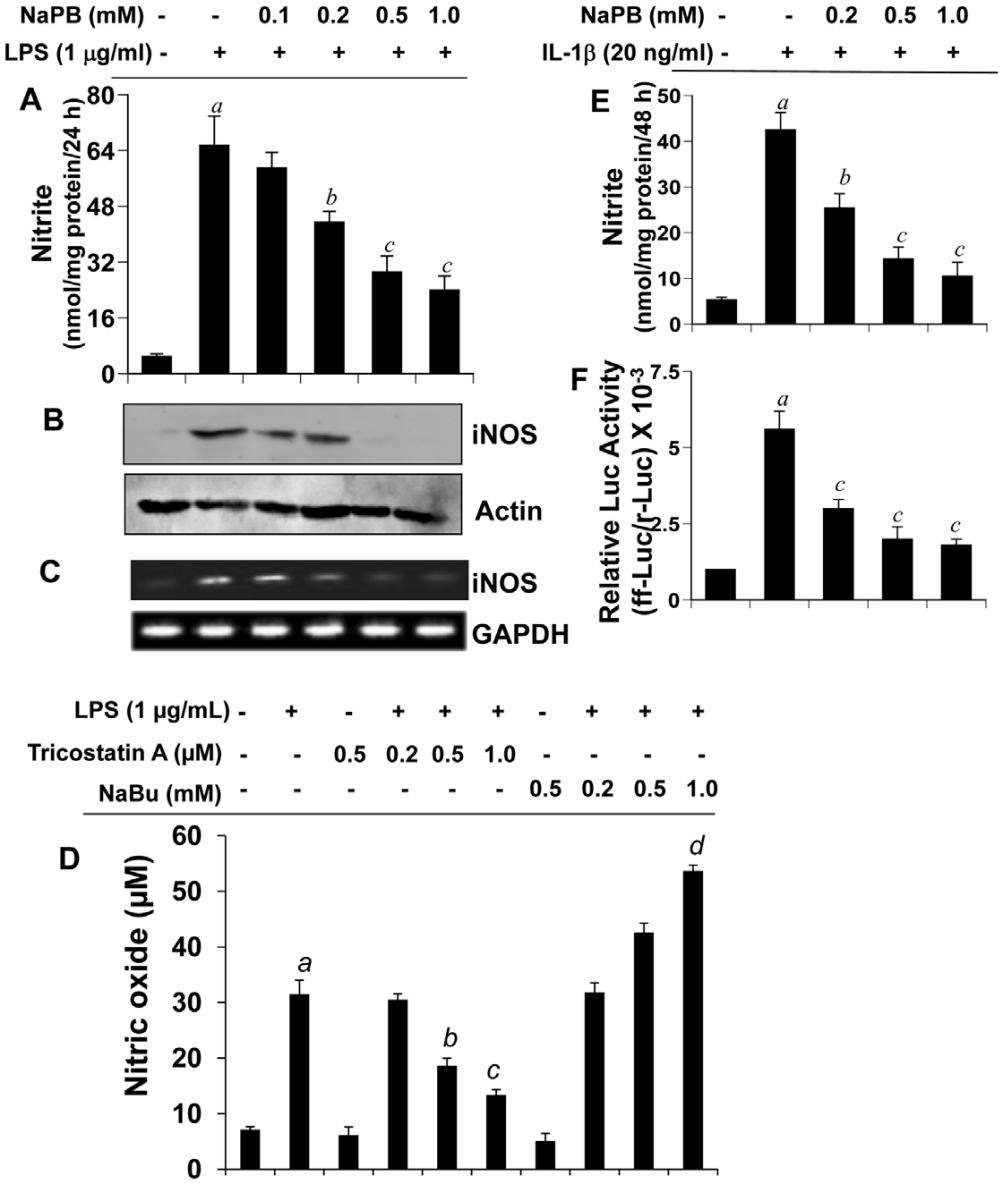
Dose-dependent inhibition of NO production by NaPB in mouse and human glial cells. Primary mouse microglia were treated with different concentrations of NaPB for 6 h followed by stimulation with LPS under serum-free condition. After 24 h of stimulation, concentrations of nitrite were measured in supernatants (A) and the level of iNOS protein was monitored in cells by Western blot (B). Results are mean + SD of three different experiments. *^a^p<0.001* vs control; *^b^p<0.05* vs LPS; *^c^p<0.001* vs LPS. After 5 h of stimulation, the expression of iNOS mRNA was monitored by semi-quantitative RT-PCR (C). Cells preincubated with different concentration of trichostatin A (TSA) and sodium butyrate (NaBu) for 6 h were stimulated with LPS for 24 h under serum-free condition followed by monitoring the level of nitrite in supernatants (D). Results are mean + S.D. of three different experiments. *^a^p<0.001* vs control; *^b^p<0.05* vs LPS; *^c,d^p<0.001* vs LPS. Primary human astroglia isolated from fetal brain tissues were treated with different concentrations of NaPB for 6 h followed by stimulation with IL-1β under serum-free condition. After 48 h of stimulation, concentrations of nitrite (E) were measured in supernatants. Human astroglia plated at 70–80% confluence in 12-well plates were cotransfected with 0.25 µg of phiNOS(7.2)Luc and 12.5 ng of pRL-TK using the Lipofectamine-Plus (Invitrogen). Twenty-four h after transfection, cells received NaPB. After 6 h of incubation, cells were stimulated with IL-1β (20 ng/ml) for 12 h. Firefly (ff-Luc) and Renilla (r-Luc) luciferase activities were obtained by analyzing the total cell extract (F). Data are mean + S.D. of three different experiments. *^a^p*<0.001 versus control; *^b^p*<0.05 versus IL-1β; *^c^p*<0.001 versus IL-1β.

Activated microglia and astroglia are known to produce excessive amount of NO having the potential of damaging neurons in neurodegenerative disorders. We investigated the effect of NaPB on the expression of iNOS in microglia. LPS is a prototype inducer of various proinflammatory molecules in different cell types including mouse microglia. Therefore, primary mouse microglia preincubated with different doses of NaPB for 6 h were stimulated with LPS under serum-free condition. Although at lower concentration (100 µM), NaPB was not effective in inhibiting the production of NO, at higher concentrations (>200 µM), NaPB markedly suppressed LPS-induced production of NO in microglia (Figure 1A). However, 1 or 2 h preincubation of microglia with NaPB was not sufficient to exhibit NO inhibition (data not shown). Because NaPB is a known inhibitor of histone deacetylase (HDAC), we investigated if other inhibitors of HDAC also shared this property. We used trichostatin A (TSA) and sodium butyrate (NaBu) for this purpose. Although similar to NaPB, TSA attenuated LPS-induced production of NO, NaBu stimulated the production of NO in LPS-stimulated microglial cells (Figure 1D) suggesting that all HDAC inhibitors do not have anti-inflammatory property. To understand the mechanism for NaPB-mediated suppression of NO production, we investigated the effect of NaPB on protein and mRNA expression of iNOS in microglia. As evident from Western blot (Figure 1B) and RT-PCR (Figure 1C), NaPB dose-dependently inhibited LPS-induced protein and mRNA expression of iNOS in microglia. (3-(4,5-dimethylthiazol-2-yl)-2,5-diphenyltetrazolium bromide (MTT) results show that NaPB was not toxic to microglia at any of the concentrations tested (data not shown) suggesting that the inhibitory effect of NaPB on microglial expression of iNOS was not due to any change in cell viability. In addition to producing NO, activated microglia also secrete a broad range of proinflammatory molecules. Therefore, we examined if NaPB was capable of suppressing the expression of proinflammatory cytokines in primary mouse microglia. Similar to the inhibition of iNOS, NaPB dose-dependently inhibited the production of TNF-α and IL-1β protein in activated microglia (Table-1).

**Table 1:** Attenuation of proinflammatory cytokine production by NaPB in primary mouse microglia.

Cytokine (ng/mgprotein/24 h)	Treatment					
	Control	LPS	LPS+NaPB (0.1 mM)	LPS+NaPB (0.2 mM)	LPS+NaPB (0.5 mM)	NaPB (0.5 mM) only
TNF-α	0	18.4+3.1	16.8+1.5	12.7+0.8	8.6+1.1^a^	0
IL-1β	0	11.2+2.9	9.9+1.4	7.5+1.1	5.3+0.6^a^	0

Primary microglia preincubated with different concentrations of NaPB for 6 h were stimulated with LPS for 24 h followed by quantification of TNF-α and IL-1β in supernatants by ELISA. Results are mean + SD of three independent experiments.

a *p<0.001 vs LPS*.

Next, we examined if NaPB could suppress the expression of iNOS in human brain cells. Astroglia are the major glial cells in the CNS and astroglial activation also plays a role in various neurodegenerative disorders. Earlier we have found that IL-1β is the only cytokine that induces iNOS in primary human astroglia [23]. Consistently, IL-1β induced the production of nitrite (Figure 1E) and the activation of human iNOS promoter (Figure 1F) in primary astroglia isolated from human fetal brains. Interestingly, NaPB markedly inhibited IL-1β-induced production of NO (Figure 1E) and the activation of iNOS promoter (Figure 1F) in human astroglia.

### NaPB Inhibits Microglial Activation of NF-κB

**Figure 2:**
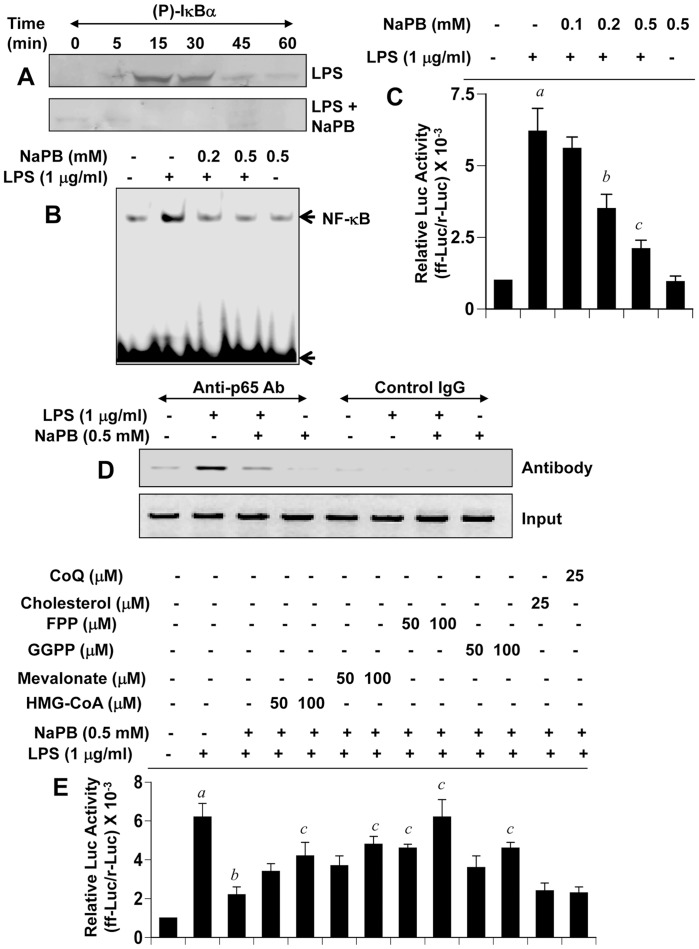
NaPB attenuates activation of NF-κB in mouse microglial cells. (A) BV-2 microglial cells preincubated with 0.5 mM NaPB for 6 h were stimulated with 1 µg/ml LPS. At different minute of stimulation, the level of phospho-IκBα was monitored by Western blot. B) Cells preincubated with different concentrations of NaPB for 6 h were stimulated with LPS for 1 h followed by monitoring the DNA-binding activity of NF-κB by EMSA. (C) Cells plated in 12-well plates were co-transfected with 0.25 µg of PBIIX-Luc (an NF-κB-dependent reporter construct) and 12.5 ng of pRL-TK. Twenty-four h after transfection, cells received different concentrations of NaPB. After 6 h of incubation, cells were stimulated with LPS for 4 h. Firefly (ff-Luc) and Renilla (r-Luc) luciferase activities were obtained by analyzing the total cell extract. Results are mean + S.D. of three different experiments. *^a^p<0.001* vs control; *^b^p<0.05* vs LPS; *^c^p<0.001* vs LPS. D) Cells preincubated with 0.5 mM NaPB for 6 h were stimulated with LPS for 2 h followed by monitoring the recruitment of RelA p65 to the mouse iNOS promoter by ChIP assay. E) Cells were co-transfected with 0.25 µg of PBIIX-Luc and 12.5 ng of pRL-TK. Twenty-four hours after transfection, cells were incubated with NaPB in the presence or absence of HMG-CoA, mevalonate, FPP, GGPP, cholesterol, and coenzyme Q. After 6 h of incubation, cells were stimulated with LPS for 4 h followed by assay of firefly (ff-Luc) and Renilla (r-Luc) luciferase activities. Results are mean + S.D. of three different experiments. *^a^p<0.001* vs control; *^b^p<0.001* vs LPS; *^c^p<0.001* vs LPS+NaPB.

LPS and other inflammatory stimuli including MPP^+^ are known to induce iNOS expression via activation of NF-κB [8], [9], [24], [25]. Because NaPB attenuated the expression of iNOS in glial cells, we examined the effect of NaPB on the activation of NF-κB. Activation of NF-κB was monitored by IκBα phosphorylation, DNA binding activity by EMSA and transcriptional activity by reporter assays. As expected, treatment of BV-2 microglial cells with LPS resulted in time-dependent increase in phospho-IκBα (Figure 2A). However, NaPB inhibited LPS-induced phosphorylation of IκBα suggesting that NaPB functions upstream of IκBα phosphorylation. Accordingly, LPS induced the DNA-binding activity of NF-κB in microglial cells, which was inhibited by NaPB (Figure 2B). We then tested the effect of NaPB on the transcriptional activity of NF-κB. As expected, LPS induced NF-κB-dependent transcription of luciferase (Figure 2C). Consistent to the effect of NaPB on the phosphorylation of IκBα and the DNA binding activity of NF-κB, NaPB also suppressed the transcriptional activity of NF-κB in a dose-dependent manner in LPS-stimulated microglia (Figure 2C).

Mouse iNOS promoter harbors two NF-κB binding sites – distal (nucleotides −971 to −962) and proximal (nucleotides −85 to −76) [26]. At first, we employed chromatin immunoprecipitation (ChIP) assay to study the recruitment of RelA p65 to each of these two NF-κB binding sites. After immunoprecipitation of LPS-stimulated microglial chromatin fragments by antibodies against p65, we were able to amplify 307 bp fragments flanking the proximal NF-κB element (Figure 2D). However, after several attempts, we failed to detect any amplification product spanning the distal NF-κB binding site (data not shown). These results suggest that LPS induced the recruitment of p65 to the proximal NF-κB-binding site of the mouse iNOS promoter in microglia. Therefore, next we examined the effect of NaPB on the recruitment of p65 to the proximal NF-κB binding site of the iNOS promoter. Consistent to the inhibition of iNOS mRNA expression, NaPB inhibited the recruitment of p65 to the iNOS promoter in LPS-stimulated microglia (Figure 2D). On the other hand, no amplification product was observed in any of the immunoprecipitates obtained with control IgG (left four lanes of Figure 2D), suggesting the specificity of these interactions. These results also suggest that NaPB interferes with the recruitment of NF-κB to the iNOS promoter.

### Intermediates of the Mevalonate Pathway Reverse the Inhibitory Effect of NaPB on Microglial NF-κB Activation

The requirement of at least 6 h of preincubation of cells with NaPB to see its anti-inflammatory effect suggests that metabolite(s) sensitive to NaPB may be involved in the process. Earlier Brahmachari and Pahan [15] have demonstrated that intermediates of the mevalonate pathway play a role in the expression of iNOS and proinflammatory cytokines in glial cells. End product of the mevalonate pathway is cholesterol and therefore, we investigated if NaPB had any effect on the level of cholesterol *in vivo* in mice. Interestingly, after 7 d of oral feeding at a dose of 200 mg/kg body wt/d, NaPB reduced the level of cholesterol in serum of mice by about 30%; and this reduction was comparable to that (∼29%) by the so-called cholesterol-lowering drug pravastatin (Figure S1). These results are important as it suggests that NaPB may be used to lower cholesterol in patients with hypercholesterolemia.

Next, we examined the role of different members of the mevalonate pathway in NF-κB inhibitory effect of NaPB. Interestingly, HMG-CoA, mevalonate, geranylgeranyl pyrophosphate (GGPP), and farnesyl pyrophosphate (FPP) abrogated the inhibitory effect of NaPB on the activation of NF-κB (Figure 2E) in microglial cells. On the other hand, cholesterol and coenzyme Q (end products of the mevalonate pathway) had no effect on NaPB-mediated inhibition of NF-κB activation (Figure 2E). These results suggest that depletion of intermediary products rather than end products of the mevalonate pathway is responsible for the observed anti-inflammatory effect of NaPB.

### NaPB Inhibits MPP^+^-induced Expression of Microglial Proinflammatory Molecules

Glial inflammation is a critical component of PD pathogenesis [2], [9], [27], [28] which is mirrored in MPTP mouse models. The neurotoxic effect of MPTP depends on its conversion into MPP^+^. In glial cells, MAO-B converts MPTP to MPP^+^, which then leads to glial activation [1]. MPP^+^ is able to stimulate the microglial expression of inflammatory molecules including NO, TNF-α, IL-1β, and IL-6 as reported by us [9], [15], [17], [29] and different other groups [30], [31]. Although, a well characterized receptor for MPP^+^ was not known in microglial cells until recent past, a new study [32] has reported that low dose of MPP^+^ stimulates microglial activation via the engagement of cysteinyl leukotriene receptor (CysLT_1_R). According to their study, MPP^+^- dependent activation of CysLT_1_R and its subsequent translocation from plasma membrane to cytosol plays a critical role in the process of microglial activation. Therefore, we investigated if MPP^+^ could induce the expression of proinflammatory molecules in microglia and if NaPB suppressed such induction. RT-PCR (Figure S2A), western blot (Figure S2B) and real-time PCR (Figure S2C) analysis showed that MPP^+^ alone induced the expression of iNOS mRNA and protein and that NaPB suppressed MPP^+^-induced iNOS mRNA and protein expression (Figure S2A–C) in microglial cells. Similarly, MPP^+^ also markedly induced the mRNA expression of IL-1β and TNF-α in microglial cells (Figure S2A, S2D & S2E). However, NaPB inhibited MPP^+^-induced expression of TNF-α and IL-1β mRNAs in a dose-dependent manner (Figure S2A, S2D & S2E) suggesting that NaPB is capable of inhibiting MPP^+^-induced expression of microglial proinflammatory molecules.

### NaPB Inhibits the Production of Reactive Oxygen Species (ROS) from Activated Microglia

**Figure 3:**
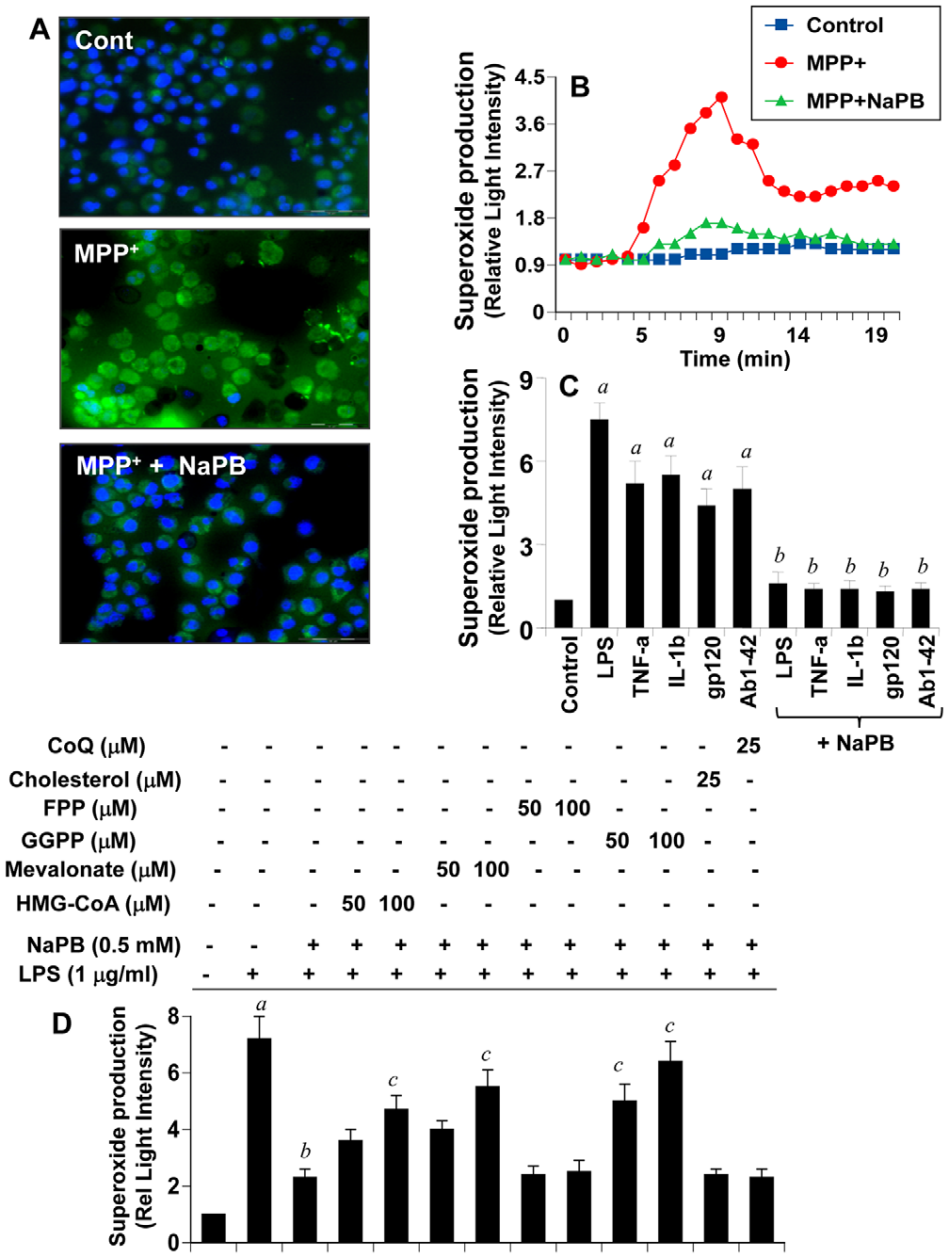
NaPB inhibits the production of ROS in mouse microglial cells. Cells were treated with 500 µM NaPB for 6 h followed by stimulation with 1 µM MPP^+^. At 15 min of stimulation, the generation of ROS was monitored by carboxy-H_2_DCFDA (A). At different intervals (measured in minutes), superoxide production was assayed in whole cells (B). Cells preincubated with 500 µM NaPB for 6 h were stimulated with LPS (1 µg/ml), TNF-α (50 ng/ml), IL-1β (20 ng/ml), gp120 (200 pg/ml), and fibrillar Aβ1-42 (1 µM). At 10 min of stimulation, superoxide production was assayed in whole cells (C). Results are mean + SD of three different experiments. *^a^p<0.001* vs control; *^b^p<0.001* vs stimuli. D) Cells were incubated with NaPB in the presence or absence of HMG-CoA, mevalonate, GGPP, FPP, cholesterol, and coenzyme Q. After 6 h of incubation, cells were stimulated with LPS for 10 min followed by assay of superoxide. Results are mean + SD of three different experiments. *^a^p<0.001* vs control; *^b^p<0.001* vs LPS; *^c^p<0.001* vs LPS+NaPB.

Oxidative stress plays an important role in the pathogenesis of various neurodegenerative diseases including PD. We examined if Parkinsonian toxin MPP^+^ induced the production of ROS from microglia and if NaPB could attenuate such ROS production. To monitor the generation of intracellular ROS in BV-2 microglial cells, we used a cell-permeant fluorescent probe. As seen in figure 3A, MPP^+^ markedly induced the generation of ROS within 15 min of stimulation. However, NaPB strongly inhibited MPP^+^-induced production of intracellular ROS (Figure 3A). Recent studies identify NADPH oxidase as the most important ROS (superoxide radicals)-producing molecule in response to different inflammatory stimuli [3], [4], [33], [34]. Accordingly, MPP^+^ also induced the production of superoxide from microglial cells in a time-dependent manner (Figure 3B). The production of superoxide was observed as early as 5 min of stimulation, which peaked at 10 min (Figure 3B). Consistent to the inhibition of endogenous ROS (Figure 3A), NaPB attenuated MPP^+^-induced production of superoxide (Figure 3B). Because various stimuli and neurotoxins are capable of producing ROS, we examined if NaPB could suppress the production of ROS in response to different inflammatory stimuli. As expected, LPS, TNF-α, IL-1β, HIV-1 gp120, and fibrillar Aβ1-42 peptides induced the production of superoxide in microglial cells (Figure 3C). However, NaPB knocked down LPS-, TNF-α-, IL-1β-, gp120-, and Aβ-induced production of superoxide in microglia (Figure 3C). These results suggest that NaPB could be used as an antioxidant.

Because NaPB exhibited anti-inflammatory activity via modulation of mevalonate metabolites (Figure 2E), we investigated if mevalonate metabolites could reverse the antioxidant effect of NaPB. Interestingly, HMG-CoA, mevalonate and GGPP, but not FPP, abolished the inhibitory effect of NaPB on the production of superoxide in microglial cells (Figure 3D). On the other hand, cholesterol and coenzyme Q had no effect on NaPB-mediated inhibition of superoxide production (Figure 3D). These results suggest that depletion of intermediary products rather than end products of the mevalonate pathway is also responsible for the antioxidant effect of NaPB. Furthermore, due to that geranylgeranylation is required for the activation of p21^rac^ and that farnesylation is needed for the activation of p21^ras^, these results suggest that p21^rac^, but not p21^ras^, may be involved in ROS generation.

### NaPB Mediates its Anti-inflammatory and Antioxidant Effects through Inhibition of p21^ras^ and p21^rac^ Activation

**Figure 4:**
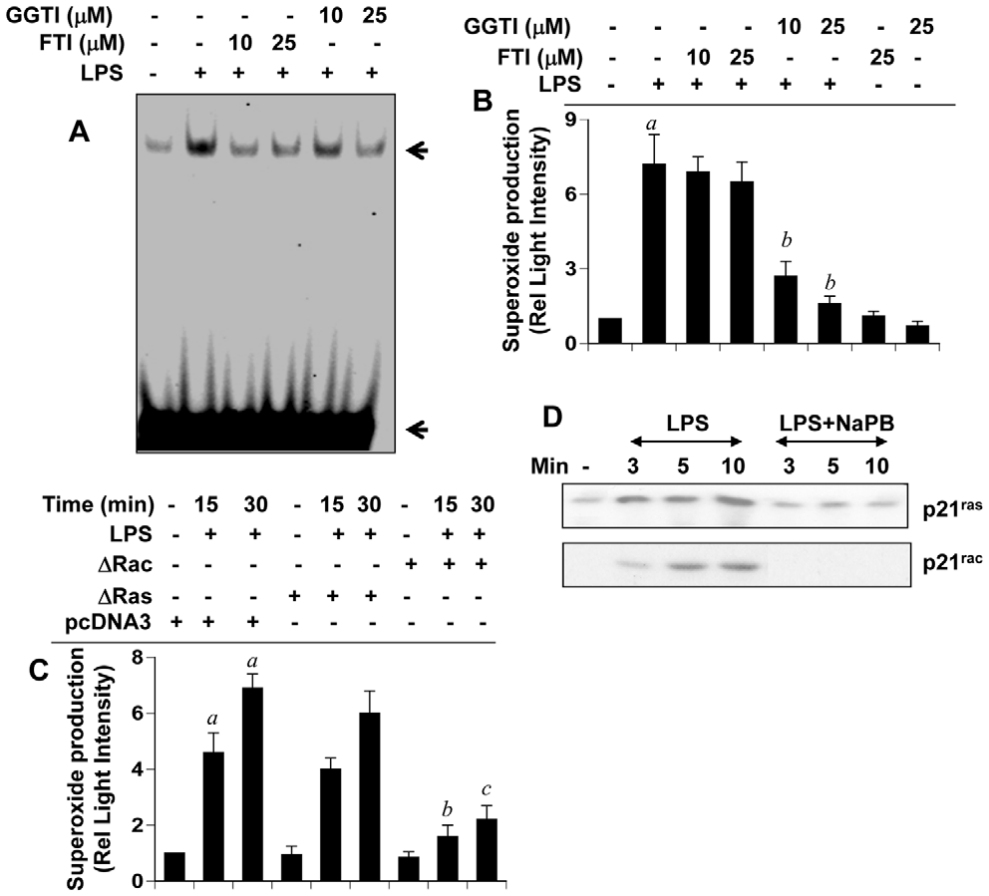
NaPB inhibits the activation of NF-κB and the production of ROS via modulation of p21^ras^ and p21^rac^. A) Mouse BV-2 microglial cells preincubated with different concentrations of FTI and GGTI were stimulated with LPS for 60 min followed by monitoring the activation of NF-κB by EMSA. B) Cells preincubated with different concentrations of FTI and GGTI were stimulated with LPS for 10 min followed by monitoring the production of superoxide. Results are mean + SD of three different experiments. *^a^p<0.001* vs control; *^b^p<0.001* vs LPS. C) Cells were transfected with Δp21^ras^, Δp21^rac^ or an empty vector using Lipofectamine Plus. Twenty-four hour after transfection, cells were stimulated with LPS followed by monitoring superoxide production at 15 and 30 min of stimulation. *^a^p<0.001* vs control; *^b^p<0.001* vs LPS-15 min; *^c^p<0.001* vs LPS-30 min. D) Cells preincubated with 500 µM NaPB were stimulated with LPS. At different time points, activation of p21^ras^ and p21^rac^ was monitored. Results represent three independent experiments.

**Figure 5:**
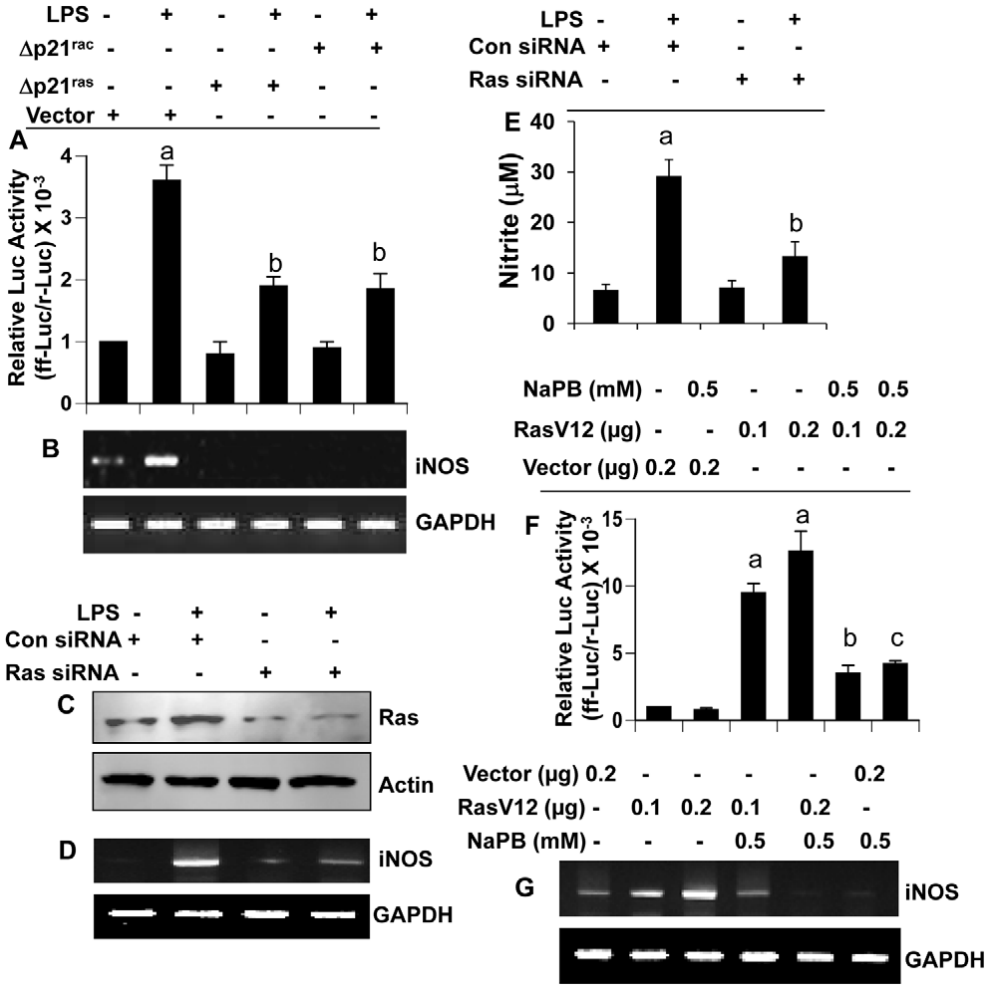
Role of p21^ras^ and p21^rac^ in the induction of iNOS and the activation of NF-κB in microglial cells. A) Cells plated in 12-well plates were co-transfected with 0.2 µg PBIIX-Luc and 0.2 µg Δp21^ras^, Δp21^rac^ or an empty vector. Each transfection also included 12.5 ng of pRL-TK. Twenty-four hour after transfection, cells were stimulated with LPS for 4 h. Firefly (ff-Luc) and Renilla (r-Luc) luciferase activities were obtained by analyzing the total cell extract. Results are mean + S.D. of three different experiments. *^a^p<0.001* vs control; *^b^p<0.001* vs LPS. B) Cells were transfected with Δp21^ras^, Δp21^rac^ or an empty vector. Twenty-four hour after transfection, cells were stimulated with LPS for 5 h under serum-free condition followed by monitoring the expression of iNOS mRNA by RT-PCR. Cells were transfected with either control siRNA or Ras siRNA and 24 h after transfection, cells were stimulated with LPS for 5 h followed by monitoring the expression of Ras protein by Western blot (C) and iNOS mRNA by RT-PCR (D). After transfection, cells were stimulated with LPS for 24 h followed by monitoring the level of nitrite in supernatants (E). *^a^p<0.001* vs control; *^b^p<0.001* vs LPS. F) Cells plated in 12-well plates were co-transfected with 0.2 µg PBIIX-Luc and different amounts of either RasV12 (a constitutively-active mutant of p21^ras^) or control vector. Each transfection also included 12.5 ng of pRL-TK. Twenty-four hour after transfection, cells were treated with NaPB for 6 h under serum-free condition followed by monitoring firefly (ff-Luc) and Renilla (r-Luc) luciferase activities in total cell extract. G) Under similar condition, the effect of RasV12 and an empty vector on the expression of iNOS mRNA was monitored in cells. Results are mean + S.D. of three different experiments. *^a^p<0.001* vs empty vector (0.2 µg); *^b^p<0.001* vs RasV12 (0.1 µg); *^c^p<0.001* vs RasV12 (0.2 µg).

Reversal of anti-inflammatory and antioxidant effects of NaPB by intermediates, but not end products, of the mevalonate pathway, suggest a possible involvement of farnesylation and/or geranylgeranylation reactions in inflammation and oxidative stress. Because farnesylation and geranylgeranylation are required for activation of p21^ras^ and p21^rac^, respectively, we examined the effect of farnesyltransferase inhibitor (FTI) and geranylgeranyltransferase inhibitor (GGTI) on NF-κB activation and superoxide production in microglial cells. Inhibition of LPS-induced activation of NF-κB by both FTI and GGTI (Figure 4A) suggests that both p21^ras^ and p21^rac^ could be involved in microglial activation of NF-κB. To confirm the involvement of p21^ras^ and p21^rac^, we examined the effect of dominant-negative mutants of p21^ras^ (Δp21^ras^) and p21^rac^ (Δp21^rac^) on LPS-induced activation of NF-κB and expression of iNOS. LPS induced the transcriptional activity of NF-κB (Figure 5A) and the expression of iNOS mRNA (Figure 5B) in empty vector-transfected microglial cells. However, both Δp21^ras^ and Δp21^rac^ suppressed LPS-induced activation of NF-κB (Figure 5A) and expression of iNOS (Figure 5B). This was further corroborated by siRNA knockdown of p21^ras^. Ras siRNA decreased the protein expression of p21^ras^ (Figure 5C) and inhibited LPS-induced expression of iNOS mRNA (Figure 5D) and production of nitrite (Figure 5E). Accordingly, RasV12, a constitutively-active mutant of p21^ras^, alone induced the activation of NF-κB (Figure 5F) and the expression of iNOS mRNA (Figure 5G) suggesting that activation of p21^ras^ alone is sufficient for the activation of NF-κB and the expression of iNOS. NaPB, in this instance as well, attenuated RasV12-induced activation of NF-κB and expression of iNOS (Figure 5F–G).

Although both FTI and GGTI inhibited the activation of NF-κB, only GGTI, but not FTI, inhibited the production of superoxide from LPS-stimulated microglial cells (Figure 4B) suggesting that p21^rac^, but not p21^ras^, could be involved in the generation of superoxide. To further confirm this finding, we examined the effect of dominant-negative mutants of p21^ras^ (Δp21^ras^) and p21^rac^ (Δp21^rac^) on LPS-induced production of superoxide. Consistently, Δp21^rac^, but not Δp21^ras^, knocked down LPS-induced production of superoxide in microglial cells. This was not seen in empty vector (pcDNA3) controls (Figure 4C). Next, we examined the effect of NaPB on the activation of p21^ras^ and p21^rac^. Marked activation of p21^ras^ and p21^rac^ was observed within minutes of LPS stimulation (Figure 4D). However, NaPB markedly suppressed LPS-induced activation of both p21^ras^ and p21^rac^ in microglial cells (Figure 4D). These results suggest that NaPB attenuates the expression of proinflammatory molecules and the production of ROS in microglia probably by suppressing the activation of p21^ras^ and p21^rac^.

### Oral Administration of NaPB Attenuates the Activation of p21^ras^ and p21^rac^ in vivo in the Nigra of MPTP-intoxicated Mice

**Figure 6:**
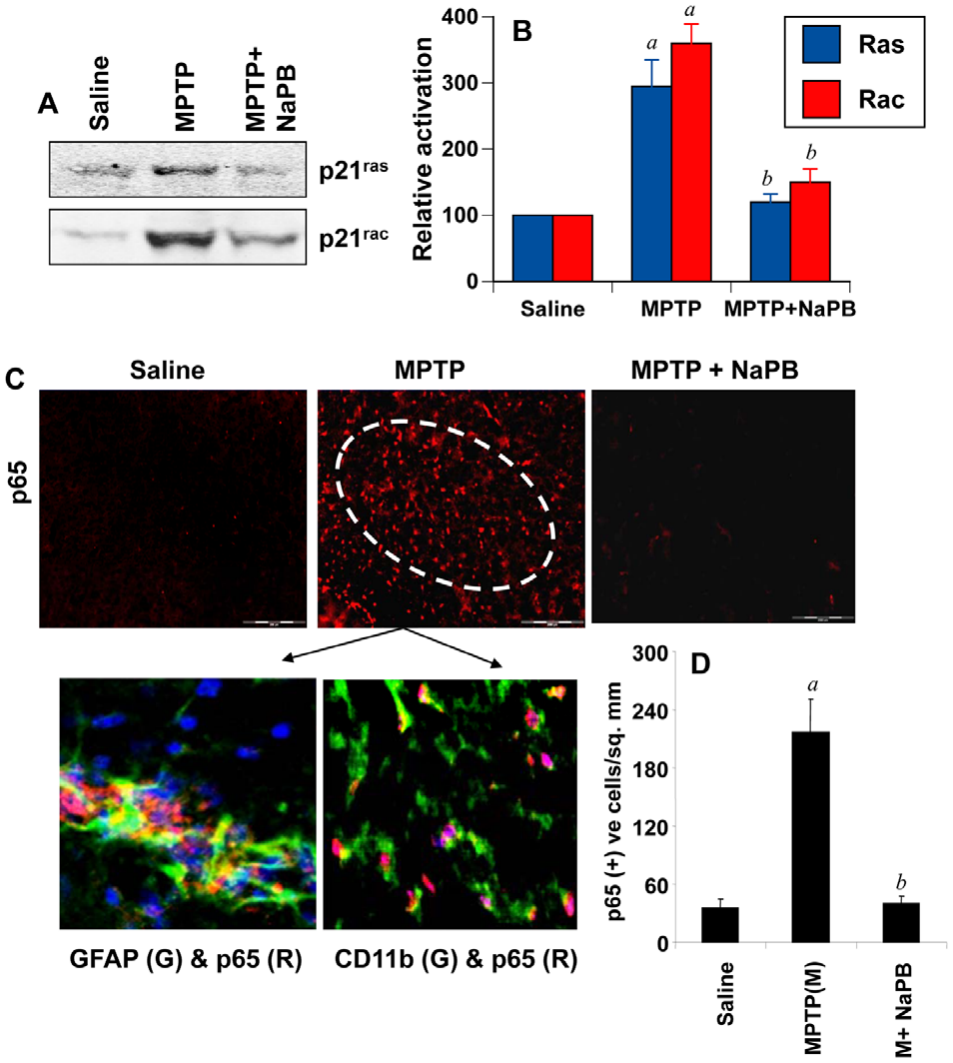
Activation of small G proteins (p21^ras^ and p21^rac^) and NF-κB in ventral midbrain of MPTP-intoxicated mice is NaPB-sensitive. A) Mice were treated with NaPB (200 mg/kg body wt/d) via gavage from 1 d prior to MPTP injection. Six h after the last injection of MPTP, activation of p21^ras^ and p21^rac^ was monitored in ventral midbrain tissues. Experiment was repeated three times each time using two animal in each group. B) Bands from three different mice were quantified and activation of p21^ras^ and p21^rac^ is shown as percent of control. C) Mice were treated with NaPB (200 mg/kg body wt/d) from 3 h after the last injection of MPTP. Twenty-four h after the last injection of MPTP, ventral midbrain sections were immunostained for p65 (low magnification). Midbrain sections of MPTP-intoxicated mice were also double-labeled for p65 and glial cell markers (GFAP for astrocytes and CD11b for microglia). Results represent three independent experiments. D) NF-κB p65 positive cells counted in four nigral sections (two images per slide) from each of four mice in an Olympus IX81 fluorescence microscope using the MicroSuite™ imaging software are mentioned as cells/mm^2^. *^a^p<0.0001* vs saline-control; *^b^p<0.0001* vs MPTP.

Because NaPB inhibits the activation of p21^ras^ and p21^rac^ in microglia, we examined if NaPB was capable of suppressing the activation of these small G proteins *in vivo* in the nigra of MPTP-insulted mice, an animal model of PD. It is clearly evident from figure 6A & 6B that MPTP intoxication markedly induced the activation of p21^ras^ and p21^rac^ in the nigra compared to saline treatment. However, mice that were treated with NaPB (200 mg/kg body wt/day) through gavage from 1 d prior to the MPTP intoxication exhibited much decreased activation of both p21^ras^ and p21^rac^ (Figure 6A & 6B) suggesting that oral NaPB is capable of inhibiting the activation of p21^ras^ and p21^rac^ *in vivo* in the nigra.

### NaPB Inhibits the Activation of NF-κB in vivo in the Nigra of MPTP-intoxicated Mice

Because NaPB inhibited the activation of NF-κB in glial cells (Figure 2), we examined if NaPB was capable of suppressing the activation of NF-κB *in vivo* in the nigra of MPTP-intoxicated mice. It is clearly evident from figure 6C and 6D that MPTP intoxication markedly induced the expression of RelA p65 in the SNpc as compared to saline treatment. Double-label immunofluorescence analysis indicates that p65 was principally expressed in CD11b-positive microglia and GFAP-positive astroglia (Figure 6C). Next, mice were treated with NaPB (200 mg/kg body wt/day) via gavage from 3 h after the last injection of MPTP and the activation of NF-κB was examined 24 h after the last injection of MPTP. As evident from figure 6C, NaPB markedly inhibited the level of p65 *in vivo* in the SNpc of MPTP-intoxicated mice.

### NaPB Inhibits the Expression of Proinflammatory Molecules in vivo in the Nigra of MPTP-intoxicated Mice

**Figure 7:**
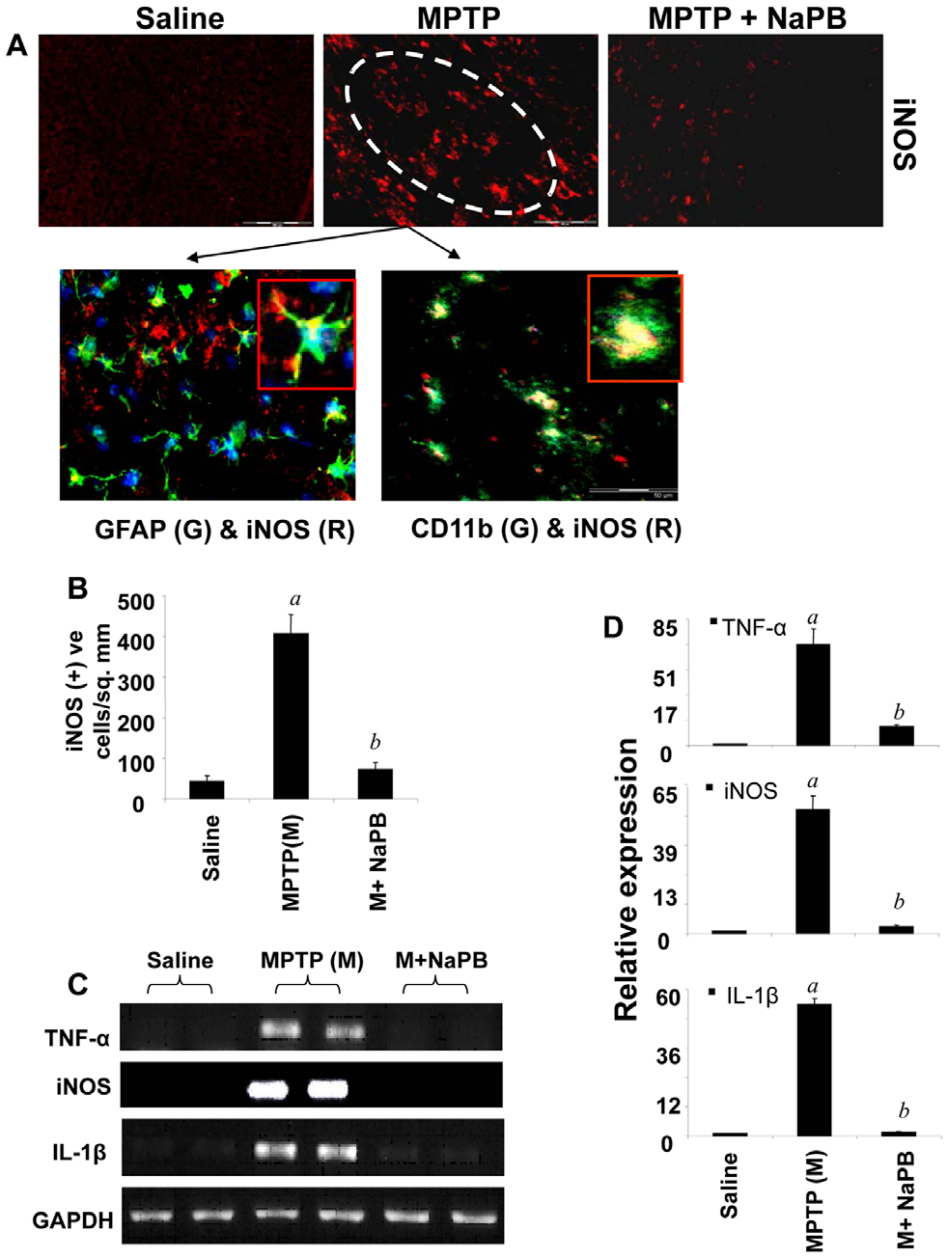
Expression of proinflammatory molecules in ventral midbrain of MPTP-intoxicated mice is NaPB-sensitive. A) Mice were treated with NaPB (200 mg/kg body wt/d) from 3 h after the last injection of MPTP. Twenty-four h after the last injection of MPTP, ventral midbrain sections were immunostained for iNOS (low magnification). Midbrain sections of MPTP-intoxicated mice were also double-labeled for iNOS and glial cell markers (GFAP for astrocytes and CD11b for microglia). Results represent three independent experiments. B) Cells positive for iNOS were counted in four nigral sections (two images per slide) from each of four mice. *^a^p<0.0001* vs saline-control; *^b^p<0.0001* vs MPTP. The mRNA expression of TNF-α, iNOS and IL-1β was analyzed by semi-quantitative RT-PCR (C) and quantitative real-time PCR (D). Data are means + SEM of five mice per group. *^a^p<0.0001* vs saline group; *^b^p<0.0001* vs the MPTP group.

Inflammation plays a role in the loss of dopaminergic neurons in PD and its animal model [9], [17], [35], [36]. Because NaPB inhibited the expression of proinflammatory molecules in glial cells and suppressed the activation of small G proteins (p21^ras^ and p21^rac^) and NF-κB *in vivo* in the nigra of MPTP-intoxicated mice, we examined if NaPB was able to suppress the expression of iNOS *in vivo* in the SNpc of MPTP-insulted mice. Immunofluorescence analysis for iNOS in ventral midbrain sections shows that MPTP intoxication led to marked increase in nigral iNOS protein expression and that iNOS co-localized with GFAP-positive astroglia and CD11b-positive microglia (Figure 7A). However, oral administration of NaPB suppressed MPTP-induced expression of iNOS protein (Figure 7A–B). As shown by semi-quantitative RT-PCR (Figure 7C) and quantitative real-time PCR (Figure 7D) experiments, MPTP intoxication led to marked increase in mRNA expression of iNOS, IL-1β and TNF-α in the nigra. However, NaPB strongly inhibited MPTP-induced expression of these proinflammatory molecules *in vivo* in the nigra (Figure 7C–D). These results suggest NaPB can inhibit the expression of proinflammatory molecules *in vivo* in the SNpc of MPTP-intoxicated mice.

### Are the Activation of p21^ras^ and the Expression of iNOS in the Nigra of MPTP-intoxicated Mice Dependent on the Loss of Dopaminergic Neurons?

Although Przedborski and colleagues [37] have shown that microglial activation occurs much earlier to neuronal death in MPTP mouse model, according to Gao et al [38], activation of glia, specifically microglia, is secondary to neurodegeneration. Therefore, in order to delineate whether glial activation-associated events happen before or after the loss of dopaminergic neurons, we performed a time-course study to monitor nigral activation of p21^ras^ and expression of iNOS and striatal loss of dopamine in MPTP-intoxicated mice. Micropunches from the nigra were used for monitoring p21^ras^ and iNOS. The activation of p21^ras^ began as early as 3 h after the last injection of MPTP, peaked at 6 h and decreased afterwards until the duration (24 h) of the study (Figure S3A). On the other hand, the expression of iNOS mRNA as revealed by RT-PCR (Figure S3B) and real-time PCR (Figure S3C) was visible as early as 6 h and maximum at 12 h. As expected from results above, NaPB treatment markedly inhibited the activation of p21^ras^ and the expression of iNOS mRNA *in vivo* the nigra at all time points tested (Figure S3A–C). In contrast to the activation of p21^ras^ and the expression of iNOS, we did not notice any loss of striatal dopamine within 24 h of MPTP intoxication (Figure S3D). Significant loss of dopamine was observed on day 5 and it was maximum on day 7 (Figure S3D). These results suggest that glial inflammation/activation appears before neuronal loss in MPTP mouse model of PD.

### NaPB Inhibits the Activation of Glial Cells in vivo in the Nigra of MPTP-intoxicated Mice

Recently activation of glial cells is being considered as a pathological hallmark in PD and other neurodegenerative disorders. Increased expression of CD11b, the beta-integrin marker of microglia, represents microglial activation during neurodegenerative inflammation (18). Similarly, upon activation, astrocytes also express enhanced level of GFAP, which is considered as a marker protein for astrogliosis [9], [39]. We investigated if NaPB could attenuate MPTP-induced activation of glial cells *in vivo* in the nigra of mice. As evident from immunofluorescence analyses of CD11b and GFAP in ventral midbrain sections (Figure S4A), MPTP intoxication led to marked increase in nigral CD11b and GFAP protein expression. However, oral treatment of MPTP-intoxicated mice with NaPB led to the inhibition of GFAP and CD11b protein expression (Figure S4A). It is clearly evident from semi-quantitative RT-PCR in Figure S4B and real-time PCR in Figure S4C that MPTP intoxication led to marked increase in mRNA expression of both CD11b and GFAP in the nigra. However, similar to the inhibition of proinflammatory molecules, NaPB suppressed MPTP-induced expression of CD11b and GFAP *in vivo* in the nigra (Figure S4B–C). These results suggest that NaPB is capable of attenuating glial activation *in vivo* in the nigra of MPTP-intoxicated mice.

### NaPB Protects Against MPTP-induced Neurodegeneration

**Figure 8:**
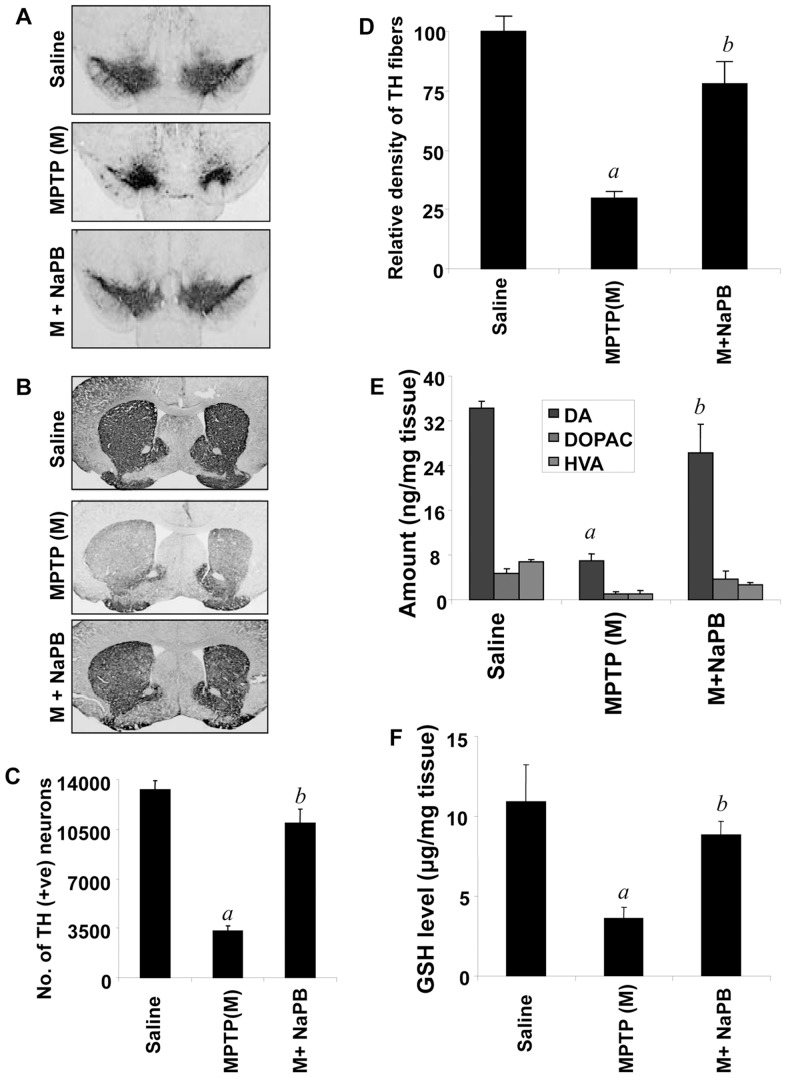
NaPB protects dopaminergic neurons in MPTP-intoxicated mice. Mice receiving NaPB (200 mg/kg body wt/day) from 3 h after the last injection of MPTP were sacrificed 7 d after the last injection of MPTP followed by TH immunostaining of SNpc (A) and striatum (B), counting of TH-positive neurons in SNpc (C), quantification of TH-positive fibers in striatum (D), assay of neurotransmitters in striatum (E), and quantification of GSH in nigra (F). Data are means + SEM of eight mice per group. *^a^p<0.0001* vs saline group; *^b^p<0.0001* vs the MPTP group.

MPTP-intoxication led to approximately 75% loss of SNpc TH-positive neurons (Figure 8A & C) and 70% reduction of striatal TH ODs (Figure 8B & D) compared with saline-injected controls. However, in MPTP-injected mice treated with NaPB, less reduction in SNpc TH-positive neurons and striatal TH ODs was observed (Figure 8A–D). Next to determine whether NaPB protects against biochemical deficits caused by MPTP, we quantified levels of dopamine (DA) and two of its metabolites, dihydroxyphenylacetic acid (DOPAC) and homovanillic acid (HVA), in the striata 7 days after the MPTP treatment. As evident from figure 8E, MPTP intoxication led to about 76% decrease in striatal DA compared to striata of saline-injected mice. In contrast, MPTP-intoxicated animals that received NaPB showed only 20–25% decrease in striatal dopamine (Figure 8E).

Because NaPB inhibited the activation of p21^rac^ and the production of ROS from microglial cells and attenuated the activation of p21^rac^ *in vivo* in the nigra of MPTP-insulted mice, we examined the effect of NaPB on nigral redox state in MPTP-insulted mice. Reduced glutathione (GSH) is the master anti-oxidant, which protects all cells including dopaminergic neurons from oxidative attack. We monitored the level of nigral GSH by HPLC. As expected, MPTP intoxication led to approximately 72% loss of GSH (Figure 8F). However, after NaPB treatment, MPTP-intoxicated mice showed only 18% loss of nigral GSH (Figure 8F) suggesting that NaPB is capable of improving the nigral redox state in MPTP-intoxicated mice.

### NaPB Improves Locomotor Functions in MPTP-intoxicated Mice

The ultimate therapeutic goal of neuroprotection is to decrease functional impairment. Therefore, to examine whether NaPB protects not only against structural and neurotransmitter damage but also against functional deficits caused by MPTP, we monitored locomotor and open-field activities. As reported earlier [17], [19], [29], MPTP injection caused marked decrease in rotorod performance (Figure S5A), movement time (Figure S 5B), total distance (Figure S5C), stereotypy (Figure S5E), rearing (Figure S5F), and horizontal activity (Figure S5G). On the other hand, MPTP increased the rest time (Figure S5D). Interestingly, NaPB significantly improved MPTP-induced hypolocomotion (Figure S5A–G).

### Is Inhibition of p21^ras^ Farnesylation and/or p21^rac^ Geranylgeranylation Sufficient to Protect the Nigrostriatum Against MPTP Toxicity?

**Figure 9:**
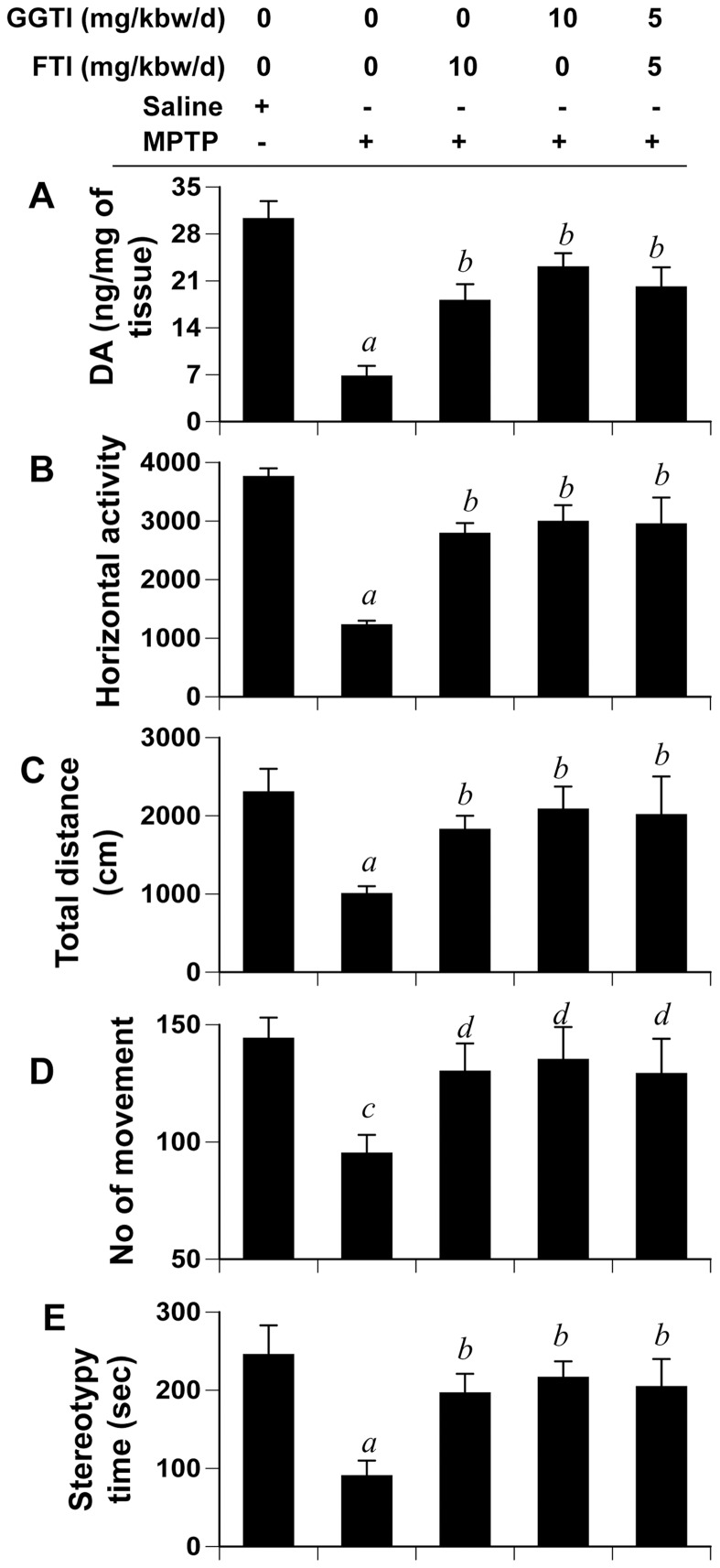
FTI and GGTI protect DA and improve locomotor activities in MPTP-intoxicated mice. Mice receiving FTI, GGTI or the combination of the two via daily intraperitoneal injection from 3 h after the last injection of MPTP were tested for motor functions (B, horizontal activity; C, total distance; D, number of movement; E, stereotypy) 7 d after the last injection of MPTP followed by measuring DA in striatum (A). Data are means + SEM of eight mice per group. *^a^p<0.001 vs saline; ^b^p<0.001 vs MPTP; ^c^p<0.05 vs saline; ^d^p<0.05 vs MPTP.*

In addition to inhibiting farnesylation of p21^ras^ and geranylgeranylation of p21^rac^, NaPB exhibits many other biological functions including histone deacetylase (HDAC) inhibition [40], chemical chaperoning upon endoplasmic reticulum (ER) stress [41] and ammonia scavenging in urea cycle disorders [42], which could be responsible for the protection of nigrostriatum from MPTP neurotoxicity. Therefore, we investigated if inhibition of either p21^ras^ or p21^rac^ alone was sufficient for the protection of the nigrostriatum. MPTP-intoxicated mice received FTI and GGTI, either separately at 10 mg/kg body wt/d or together at 5 mg/kg body wt/d, via i.p. injection from 3 h after the last injection of MPTP. After 7 d of MPTP intoxication, motor tasks were assayed followed by monitoring the level of DA in the striatum. FTI and GGTI, alone or in combination, were capable of improving horizontal activity (Figure 9B), total distance traveled (Figure 9C), number of movements (Figure 9D), and stereotypy time (Figure 9E), and reversing the loss of DA (Figure 9A) significantly in MPTP-intoxicated mice suggesting that suppression of either p21^ras^ farnesylation or p21^rac^ geranylgeranylation alone is sufficient for the protection of the nigrostriatum in MPTP-intoxicated mice.

### Does NaPB Halt the Disease Progression in a Chronic MPTP Mouse Model?

**Figure 10:**
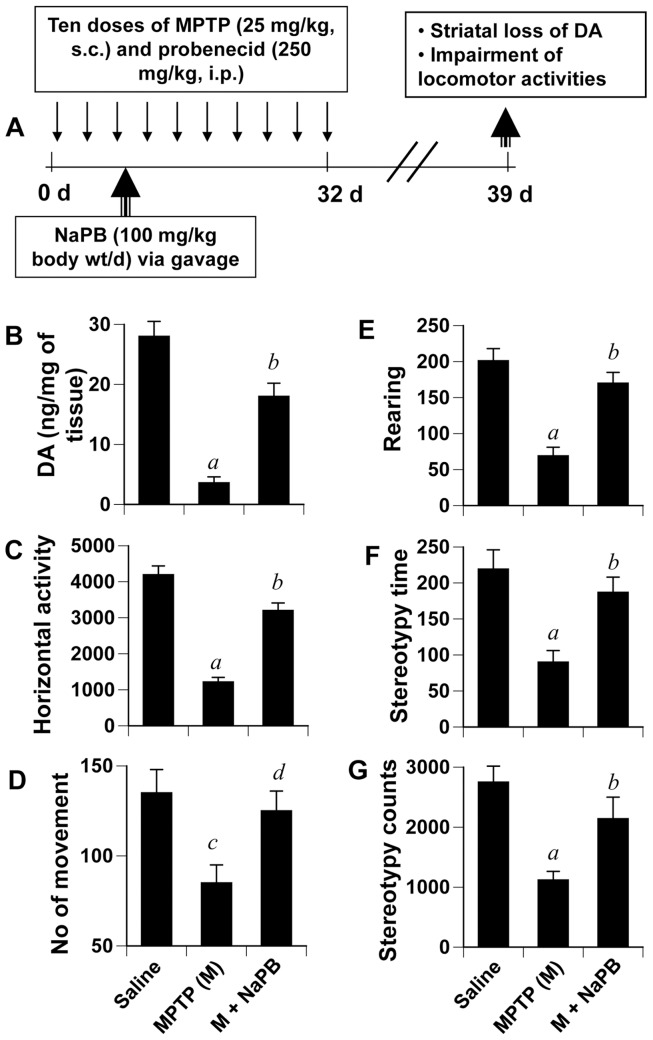
NaPB protects striatal dopamine and improves motor functions in a chronic MPTP mouse model of PD. A) Six to eight week old male C57BL/6 mice received 10 injections of MPTP (s.c.; 25 mg/kg body weight) together with 10 injections of probenecid (i.p.; 250 mg/kg body weight) for 5 weeks. Control group of mice received only saline. One group of mice were treated with NaPB (100 mg/kg body weight/d) via gavage from the 3^rd^ injection of MPTP/probenecid and continued for 1 week thereafter. Mice were tested for different motor tasks [horizontal activity (C), number of movements (D), rearing (E), and stereotypy (F)] one week after the last injection of MPTP followed by measuring the level of DA in striatum (B). Data are means + SEM of eight mice per group. *^a^p<0.001 vs saline; ^b^p<0.001 vs MPTP; ^c^p<0.05 vs saline; ^d^p<0.05 vs MPTP.*

While the acute MPTP model is helpful for quick drug screening, elucidating molecular mechanisms and determining the interaction between drug and MPTP, effects of acute intoxication reverse over time. Therefore, recently a chronic intoxicated model has been described [18], [19], in which significant loss of dopaminergic neurons is seen even six months after MPTP intoxication. We examined if NaPB was capable of protecting neurons in a chronic model (Figure 10A). As expected, chronic MPTP intoxication led to marked loss of striatal dopamine (Figure 10B) and decrease in horizontal activity (Figure 10C), number of movements (Figure 10D), rearing (Figure 10E), and stereotypy (Figure 10F–G). We initiated oral NaPB treatment (100 mg/kg body wt/d) from the third injection of MPTP/probenecid (Figure 10A). Consistent to that observed in the acute model (Figure 8), NaPB, in this instance, also significantly protected striatal DA (Figure 10B and improved locomotor activities (Figure 10C–G).

## Discussion

Neuroinflammation and oxidative stress are two hallmarks of neurodegenerative disorders. Therefore, identifying drugs to attenuate the production of proinflammatory molecules and ROS is an important area of research as such drugs may stop or delay the progression of neurodegenerative disorders. Sodium phenylbutyrate (NaPB) is a sodium salt of short chain fatty acid having multiple clinical interests. Being known as Buphenyl® or triButyrate® in the US or Ammonaps® in Sweden, it is used as a medication against urea cycle disorders involving deficiencies of carbamylphosphate synthetase, ornithine transcarbamylase, or argininosuccinic acid synthetase. Although its major function is to scavenge ammonia and glutamine [42], recent studies have described NaPB as a potent inhibitor of HDAC [40]. Due to its HDAC inhibitory effects, it has been proposed as a drug against various forms of cancer [43]. Accordingly, it has been also clinically tested as an anticancer drug [44]. In addition to ammonia removal and HDAC inhibition, Ozcan et al [41] have shown that NaPB can also function as a chemical chaperon during endoplasmic reticulum (ER) stress. Here we delineate two new functions of NaPB. On one hand, NaPB inhibits the expression of various proinflammatory molecules (iNOS, TNF-α and IL-1β) from activated glial cells suggesting that NaPB is anti-inflammatory. On the other, it suppresses the production of ROS from microglial cells in response to various stimuli indicating its antioxidant activities.

The signaling events required for the transcription of iNOS and proinflammatory cytokines are becoming clear. Although many transcription factors such as NF-κB, C/EBPβ, AP-1, STAT, IRF-1 etc play a role, activation of NF-κB seems essential for the transcription of most of the proinflammatory molecules [8], [9], [24], [25], [26], [45]. Therefore, for a drug to exhibit an anti-inflammatory effect, it is almost mandatory to attenuate the activation of NF-κB. Accordingly, NaPB inhibits the activation of NF-κB at both DNA-binding and transcriptional levels. However, it was unknown by which mechanisms NaPB suppressed the activation of NF-κB. p21^ras^ is a membrane-associated small guanine nucleotide-binding protein that plays a central role in transmitting many extracellular signals within the cell [46], [47]. Here we present evidence that NaPB suppressed the activation of p21^ras^ and thereby inhibited the activation of NF-κB in microglia. Our conclusion is based on the following observations: *First,* NaPB reduced the level of cholesterol *in vivo* in mice at a level comparable to pravastatin suggesting that this drug may be used to lower cholesterol in patients with hypercholesterolemia. However, HMG-CoA, mevalonate, GGPP, and FPP, but not cholesterol, reversed the inhibitory effect of NaPB on the activation of NF-κB suggesting that depletion of intermediates, but not end products, of the mevalonate pathway is involved in the anti-inflammatory effect of NaPB. *Second,* FTI, capable of inhibiting farnesylation of p21^ras^, and GGTI, capable of inhibiting geranylgeranylation of p21^rac^, inhibited the activation of NF-κB, suggesting the involvement of both p21^ras^ and p21^rac^ in LPS-induced microglial activation of NF-κB. *Third,* NaPB inhibited the activation of both p21^ras^ and p21^rac^ in LPS-stimulated microglial cells. These small G proteins function by binding to the cytoplasmic surface of the plasma membrane. This membrane localization involves isoprenylation of small G proteins at the C-terminus [46]. Our results suggest that NaPB attenuates farnesylation of p21^ras^ and geranylgeranylation of p21^rac^ and thereby inhibits the signal transmission to the downstream signaling molecules [46], [47]. Although here we demonstrate that NaPB inhibits the phosphorylation of IκBα thereby attenuating the activation of NF-κB, p65 is known to be acetylated by histone acetyl transferases (HAT) [48]. Therefore, being an inhibitor of HDAC, NaPB may also inhibit the acetylation of p65 and suppress its transcriptional activity.

Although there are many ROS-producing molecules, recent studies identify NADPH oxidase as the most important ROS producer in response to various inflammatory and degenerative stimuli. NADPH oxidase is a five-subunit protein that generates superoxide from molecular oxygen and is composed of two membrane-bound subunits, gp91^phox^ and p22^phox^, and at least two cytosolic subunits, p47^phox^ and p67^phox^. During its activation, p21^rac^ comes into the picture, associates with p67^phox^ and gp91^phox^ and completes the formation of the active enzyme complex [3]. Then this active NADPH oxidase catalyzes the formation of superoxide from molecular oxygen. Here, we demonstrate that NaPB suppressed the activation of p21^rac^ and thereby reduced the production of ROS from activated microglia. Our conclusion is based on the following observations: *First,* HMG-CoA, mevalonate and GGPP, but not FPP, reversed the inhibitory effect of NaPB on superoxide, suggesting that geranylgeranylation, but not farnesylation, is involved in the antioxidant effect of NaPB. *Second,* GGTI, but not FTI, inhibited the production of superoxide from activated microglia. Because GGTI inhibits geranylgeranylation of p21^rac^, these results suggest that p21^rac^, but not p21^ras^, may be involved in LPS-induced microglial production of superoxide. *Third,* a dominant-negative mutant of p21^rac^ (Δp21^rac^), but not p21^ras^ (Δp21^ras^), knocked down microglial production of superoxide. *Fourth,* NaPB inhibited the activation of p21^rac^ in LPS-stimulated microglial cells. Similar to p21^ras^, p21^rac^ also requires isoprenylation for attachment to plasma membrane. However, in contrast to farnesylation of p21^ras^, p21^rac^ requires geranylgeranylation for membrane attachment and activation. Our results suggest that NaPB suppresses geranylgeranylation of p21^rac^ and thereby inhibits its assembly with the NADPH oxidase complex.

The MPTP mouse model is particularly useful in testing new therapeutic intervention in PD. Because NaPB inhibits the production of proinflammatory molecules and ROS, hallmarks of neurodegenerative pathology, we decided to investigate the efficacy of NaPB in protecting nigrostriatal neurons in the MPTP mouse model of PD. Several lines of evidence presented in this manuscript clearly establish that NaPB is capable of protecting dopaminergic neurons from Parkinsonian toxicity. When this manuscript was in preparation, Zhou et al [49] have shown that NaPB upregulates DJ-1 in neurons and protects dopaminergic neurons in MPTP mouse model. However, here we have demonstrated that NaPB is capable of suppressing glial activation and reducing the production of proinflammatory molecules and ROS in activated glial cells and *in vivo* in the SNpc of MPTP-intoxicated mice via modulation of small G proteins.

There are several advantages of NaPB over other proposed anti-neurodegenerative therapies. *First,* NaPB is fairly nontoxic. It is an FDA-approved drug against urea cycle disorders in children. *Second,* NaPB can be taken orally, the least painful route. Here we have shown that oral administration of NaPB protects the nigrostriatum in both acute and chronic MPTP models of PD. *Third,* although here we have not tested, NaPB being a lipophilic molecule is most likely able to diffuse through the blood-brain barrier. For example, glutamine toxicity is a problem in urea cycle disorders. After treatment of patients with urea cycle disorders, NaPB is known to combine with glutamine to produce phenylacetylglutamine, a compound that is readily excreted in the urine. Simultaneous serum and CSF sampling in those patients showed comparable levels of phenylacetylglutamine in the CSF [50], suggesting that NaPB is capable of crossing the BBB.

In summary, we have demonstrated that NaPB inhibits glial activation of NF-κB by suppressing p21^ras^ activation and that NaPB attenuates ROS production by suppressing p21^rac^ activation. Because NaPB suppresses the mevalonate pathway, it is also able to lower cholesterol *in vivo* in mice at a level comparable to pravastatin, a cholesterol-lowering drug, suggesting that NaPB may be a choice of cholesterol-lowering drug in cases where statins exhibit toxicity. We also demonstrate that NaPB suppresses the activation of p21^ras^ and p21^rac^ and blocks the activation of NF-κB in the SNpc, inhibits nigral expression of proinflammatory molecules and the of activation glial cells, increases the level antioxidant GSH in the SNpc, protects the loss of dopaminergic neurons, and improves the behavioral functions in MPTP-intoxicated mice. These results highlight undiscovered properties of NaPB and indicate that NaPB may be used for therapeutic intervention in PD or neurodegenerative disorders.

## Supporting Information

Figure S1 **Effect of NaPB on serum level of cholesterol in male C57/BL6 mice.** Mice (6–8 wk old) were treated with NaPB (200 mg/kg body wt/d) and pravastatin (1 mg/kg body wt/d) separately via gavage for 7 d followed by quantification of cholesterol in serum using a simple fluorometric method. Results represent mean + SD of five mice per group (n = 5). *^a^p<0.05* vs control.
(TIF)

Figure S2 **NaPB attenuates MPP^+^-induced expression of proinflammatory molecules in mouse microglial cells.** Mouse BV-2 microglial cells were treated with different concentrations of NaPB for 6 h followed by stimulation with 1 µM MPP^+^ under serum-free condition. After 5 h of stimulation, the mRNA expression of iNOS, IL-1β and TNF-α was monitored by semi-quantitative RT-PCR (A) and quantitative real-time PCR (C, iNOS; D, IL-1β; E, TNF-α). After 24 h of stimulation, the protein expression of iNOS was monitored by Western blot (B). Results are mean + SD of three different experiments. *^a^p<0.001* vs control; *^b^p<0.001* vs MPP^+^.
(TIF)

Figure S3 **Time course of nigral activation of p21^ras^ and expression of iNOS and striatal loss of dopamine in MPTP-intoxicated mice.** A) Mice were treated with NaPB (200 mg/kg body wt/d) via gavage from 1 d prior to MPTP injection. At different h of MPTP insult, nigral punches from two mice were pooled together and analyzed for the activation of p21^ras^ (A) and the expression of iNOS mRNA (B, RT-PCR; C, real-time PCR). Experiments were repeated three times each time using two animals in each group. *^a^p<0.001 vs control (0h)-iNOS.* D) At different days of MPTP insult, dopamine was quantified in striata. Data are means + SEM of six mice per group. ^*^p<0.05 vs control; ^**^p<0.001 vs control.
(TIF)

Figure S4 **Increased expression of CD11b and GFAP in ventral midbrain of MPTP-intoxicated mice is NaPB-sensitive.** A) Mice were treated with NaPB (200 mg/kg body wt/d) from 3 h after the last injection of MPTP. Twenty-four h after the last injection of MPTP, ventral midbrain sections were immunostained for GFAP and CD11b. Results represent analyses of four nigral sections (two images per slide) from each of four mice. The mRNA expression of GFAP and CD11b was analyzed by semi-quantitative RT-PCR (B) and quantitative real-time PCR (C). Data are means + SEM of five mice per group. *^a^p<0.0001* vs saline group; *^b^p<0.0001* vs the MPTP group.
(TIF)

Figure S5 **NaPB improves motor functions in MPTP-intoxicated mice.** Mice receiving NaPB (200 mg/kg body wt/day) from 3 h after the last injection of MPTP were tested for motor functions (A, rotorod; B, movement time; C, total distance; D, rest time; E, stereotypy counts; F, rearing; G, horizontal activity) 7 d after the last injection of MPTP. Data are means + SEM of eight mice per group.*^a^p<0.001 vs MPTP; ^b^p<0.05 vs MPTP.*
(TIF)

## References

[1] Dauer W, Przedborski S (2003). Parkinson’s disease: mechanisms and models.. Neuron.

[2] Gao HM, Liu B, Zhang W, Hong JS (2003). Novel anti-inflammatory therapy for Parkinson’s disease.. Trends Pharmacol Sci.

[3] Bokoch GM, Knaus UG (2003). NADPH oxidases: not just for leukocytes anymore! Trends Biochem Sci.

[4] Wu DC, Teismann P, Tieu K, Vila M, Jackson-Lewis V (2003). NADPH oxidase mediates oxidative stress in the 1-methyl-4-phenyl-1,2,3,6-tetrahydropyridine model of Parkinson’s disease.. Proc Natl Acad Sci U S A.

[5] Zhang W, Wang T, Qin L, Gao HM, Wilson B (2004). Neuroprotective effect of dextromethorphan in the MPTP Parkinson’s disease model: role of NADPH oxidase.. Faseb J.

[6] Nagatsu T, Mogi M, Ichinose H, Togari A (2000). Changes in cytokines and neurotrophins in Parkinson’s disease.. J Neural Transm.

[7] Giulian D, Baker TJ (1986). Characterization of ameboid microglia isolated from developing mammalian brain.. J Neurosci.

[8] Roy A, Fung YK, Liu X, Pahan K (2006). Up-regulation of microglial CD11b expression by nitric oxide.. J Biol Chem.

[9] Jana M, Jana A, Liu X, Ghosh S, Pahan K (2007). Involvement of phosphatidylinositol 3-kinase-mediated up-regulation of I kappa B alpha in anti-inflammatory effect of gemfibrozil in microglia.. J Immunol.

[10] Saha RN, Jana M, Pahan K (2007). MAPK p38 regulates transcriptional activity of NF-kappaB in primary human astrocytes via acetylation of p65.. J Immunol.

[11] Jana M, Jana A, Pal U, Pahan K (2007). A simplified method for isolating highly purified neurons, oligodendrocytes, astrocytes, and microglia from the same human fetal brain tissue.. Neurochem Res.

[12] Liu X, Jana M, Dasgupta S, Koka S, He J (2002). Human immunodeficiency virus type 1 (HIV-1) tat induces nitric-oxide synthase in human astroglia.. J Biol Chem.

[13] Khasnavis S, Pahan K (2012). Sodium Benzoate, a Metabolite of Cinnamon and a Food Additive, Upregulates Neuroprotective Parkinson Disease Protein DJ-1 in Astrocytes and Neurons.. J Neuroimmune Pharmacol.

[14] Saha RN, Liu X, Pahan K (2006). Up-regulation of BDNF in astrocytes by TNF-alpha: a case for the neuroprotective role of cytokine.. J Neuroimmune Pharmacol.

[15] Brahmachari S, Jana A, Pahan K (2009). Sodium benzoate, a metabolite of cinnamon and a food additive, reduces microglial and astroglial inflammatory responses.. J Immunol.

[16] Roy A, Jana A, Yatish K, Freidt MB, Fung YK (2008). Reactive oxygen species up-regulate CD11b in microglia via nitric oxide: Implications for neurodegenerative diseases.. Free Radic Biol Med.

[17] Ghosh A, Roy A, Matras J, Brahmachari S, Gendelman HE (2009). Simvastatin inhibits the activation of p21ras and prevents the loss of dopaminergic neurons in a mouse model of Parkinson’s disease.. J Neurosci.

[18] Meredith GE, Totterdell S, Potashkin JA, Surmeier DJ (2008). Modeling PD pathogenesis in mice: advantages of a chronic MPTP protocol.. Parkinsonism Relat Disord.

[19] Roy A, Pahan K (2011). Prospects of statins in Parkinson disease.. Neuroscientist.

[20] Tieu K, Perier C, Caspersen C, Teismann P, Wu DC (2003). D-beta-hydroxybutyrate rescues mitochondrial respiration and mitigates features of Parkinson disease.. J Clin Invest.

[21] Pahan K, Liu X, McKinney MJ, Wood C, Sheikh FG (2000). Expression of a dominant-negative mutant of p21(ras) inhibits induction of nitric oxide synthase and activation of nuclear factor-kappaB in primary astrocytes.. J Neurochem.

[22] Benner EJ, Mosley RL, Destache CJ, Lewis TB, Jackson-Lewis V (2004). Therapeutic immunization protects dopaminergic neurons in a mouse model of Parkinson’s disease.. Proc Natl Acad Sci U S A.

[23] Jana M, Anderson JA, Saha RN, Liu X, Pahan K (2005). Regulation of inducible nitric oxide synthase in proinflammatory cytokine-stimulated human primary astrocytes.. Free Radic Biol Med.

[24] Jana M, Liu X, Koka S, Ghosh S, Petro TM (2001). Ligation of CD40 stimulates the induction of nitric-oxide synthase in microglial cells.. J Biol Chem.

[25] Pahan K, Sheikh FG, Namboodiri AM, Singh I (1997). Lovastatin and phenylacetate inhibit the induction of nitric oxide synthase and cytokines in rat primary astrocytes, microglia, and macrophages.. J Clin Invest.

[26] Xie QW, Kashiwabara Y, Nathan C (1994). Role of transcription factor NF-kappa B/Rel in induction of nitric oxide synthase.. J Biol Chem.

[27] Olanow CW, Tatton WG (1999). Etiology and pathogenesis of Parkinson’s disease.. Annu Rev Neurosci.

[28] Qureshi GA, Baig S, Bednar I, Sodersten P, Forsberg G (1995). Increased cerebrospinal fluid concentration of nitrite in Parkinson’s disease.. Neuroreport.

[29] Ghosh A, Roy A, Liu X, Kordower JH, Mufson EJ (2007). Selective inhibition of NF-kappaB activation prevents dopaminergic neuronal loss in a mouse model of Parkinson’s disease.. Proc Natl Acad Sci U S A.

[30] Lahaie-Collins V, Bournival J, Plouffe M, Carange J, Martinoli MG (2008). Sesamin modulates tyrosine hydroxylase, superoxide dismutase, catalase, inducible NO synthase and interleukin-6 expression in dopaminergic cells under MPP+-induced oxidative stress.. Oxid Med Cell Longev.

[31] Rojo AI, Innamorato NG, Martin-Moreno AM, De Ceballos ML, Yamamoto M (2010). Nrf2 regulates microglial dynamics and neuroinflammation in experimental Parkinson’s disease.. Glia.

[32] Wang Y, Zhang X, Li C, Zhao J, Wei E (2012). Involvement of 5-lipoxygenase/cysteinyl leukotriene receptor 1 in rotenone- and MPP+-induced BV2 microglial activation.. Mol Neurodegener.

[33] Jana A, Pahan K (2004). Fibrillar amyloid-beta peptides kill human primary neurons via NADPH oxidase-mediated activation of neutral sphingomyelinase. Implications for Alzheimer’s disease.. J Biol Chem.

[34] Jana A, Pahan K (2004). Human immunodeficiency virus type 1 gp120 induces apoptosis in human primary neurons through redox-regulated activation of neutral sphingomyelinase.. J Neurosci.

[35] Dehmer T, Lindenau J, Haid S, Dichgans J, Schulz JB (2000). Deficiency of inducible nitric oxide synthase protects against MPTP toxicity in vivo.. J Neurochem.

[36] Hunot S, Boissiere F, Faucheux B, Brugg B, Mouatt-Prigent A (1996). Nitric oxide synthase and neuronal vulnerability in Parkinson’s disease.. Neuroscience.

[37] Wu DC, Jackson-Lewis V, Vila M, Tieu K, Teismann P (2002). Blockade of microglial activation is neuroprotective in the 1-methyl-4-phenyl-1,2,3,6-tetrahydropyridine mouse model of Parkinson disease.. J Neurosci.

[38] Gao HM, Zhou H, Zhang F, Wilson BC, Kam W (2011). HMGB1 acts on microglia Mac1 to mediate chronic neuroinflammation that drives progressive neurodegeneration.. J Neurosci.

[39] Brahmachari S, Fung YK, Pahan K (2006). Induction of glial fibrillary acidic protein expression in astrocytes by nitric oxide.. J Neurosci.

[40] Butler R, Bates GP (2006). Histone deacetylase inhibitors as therapeutics for polyglutamine disorders.. Nat Rev Neurosci.

[41] Ozcan U, Yilmaz E, Ozcan L, Furuhashi M, Vaillancourt E (2006). Chemical chaperones reduce ER stress and restore glucose homeostasis in a mouse model of type 2 diabetes.. Science.

[42] Maestri NE, Brusilow SW, Clissold DB, Bassett SS (1996). Long-term treatment of girls with ornithine transcarbamylase deficiency.. N Engl J Med.

[43] Bolden JE, Peart MJ, Johnstone RW (2006). Anticancer activities of histone deacetylase inhibitors.. Nat Rev Drug Discov.

[44] Phuphanich S, Baker SD, Grossman SA, Carson KA, Gilbert MR (2005). Oral sodium phenylbutyrate in patients with recurrent malignant gliomas: a dose escalation and pharmacologic study.. Neuro Oncol.

[45] Saha RN, Pahan K (2006). Signals for the induction of nitric oxide synthase in astrocytes.. Neurochem Int.

[46] Hancock JF, Cadwallader K, Marshall CJ (1991). Methylation and proteolysis are essential for efficient membrane binding of prenylated p21K-ras(B).. Embo J.

[47] Kikuchi A, Williams LT (1994). The post-translational modification of ras p21 is important for Raf-1 activation.. J Biol Chem.

[48] Liu Y, Denlinger CE, Rundall BK, Smith PW, Jones DR (2006). Suberoylanilide hydroxamic acid induces Akt-mediated phosphorylation of p300, which promotes acetylation and transcriptional activation of RelA/p65.. J Biol Chem.

[49] Zhou W, Bercury K, Cummiskey J, Luong N, Lebin J (2011). Phenylbutyrate up-regulates the DJ-1 protein and protects neurons in cell culture and in animal models of Parkinson disease.. J Biol Chem.

[50] Thibault A, Cooper MR, Figg WD, Venzon DJ, Sartor AO (1994). A phase I and pharmacokinetic study of intravenous phenylacetate in patients with cancer.. Cancer Res.

