# Supplementary figures and images for: Sodium Phenylbutyrate Controls Neuroinflammatory and Antioxidant Activities and Protects Dopaminergic Neurons in Mouse Models of Parkinson’s Disease
Source: PLoS One. 2012 Jun 18;7(6):e38113. doi: 10.1371/journal.pone.0038113 (PMC3377667; doi:10.1371/journal.pone.0038113)

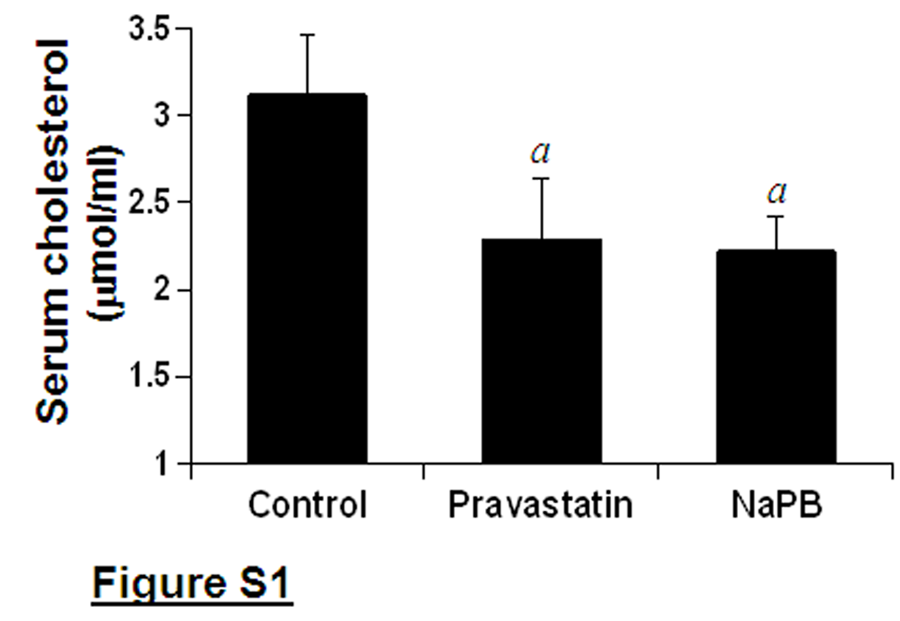

Supplement: Figure S1 — Effect of NaPB on serum level of cholesterol in male C57/BL6 mice. Mice (6–8 wk old) were treated with NaPB (200 mg/kg body wt/d) and pravastatin (1 mg/kg body wt/d) separately via gavage for 7 d followed by quantification of cholesterol in serum using a simple fluorometric method. Results represent mean + SD of five mice per group (n = 5). ap<0.05 vs control. (TIF) [file pone.0038113.s001.tif]

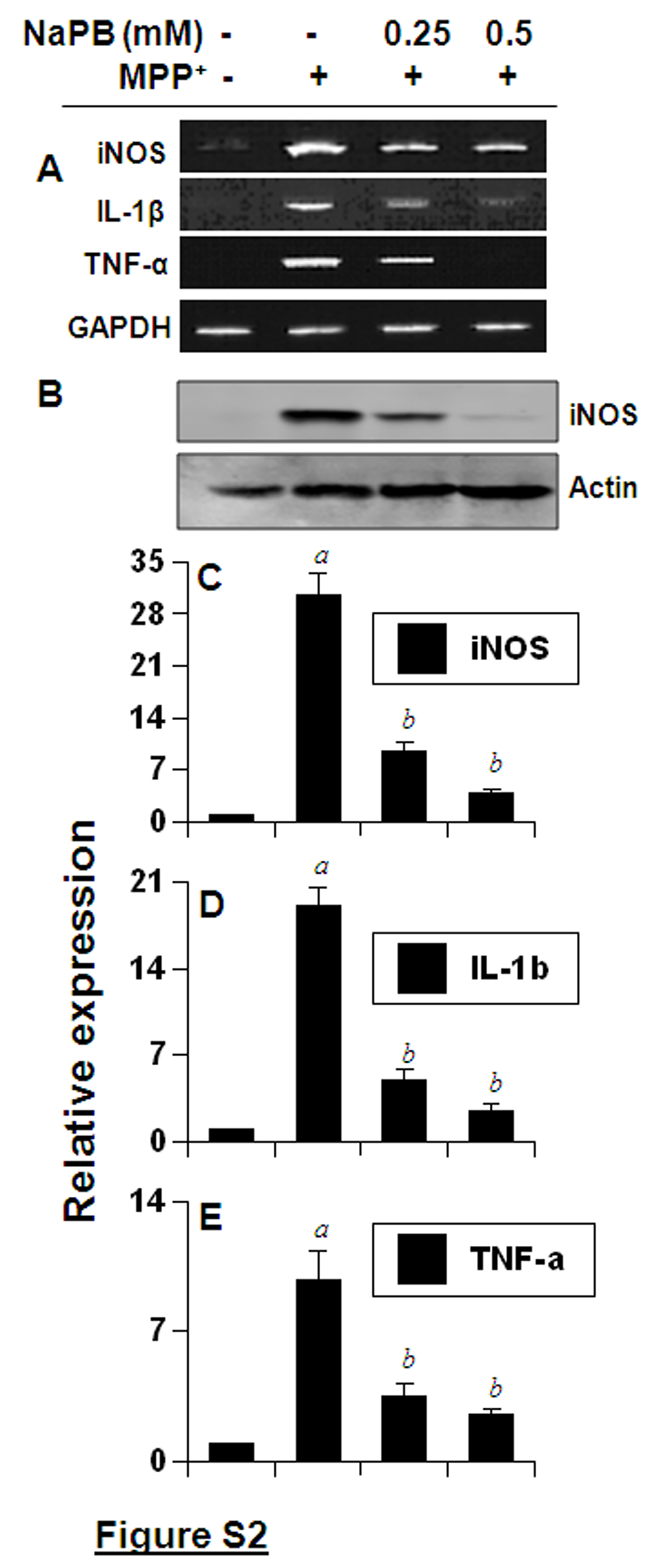

Supplement: Figure S2 — NaPB attenuates MPP+-induced expression of proinflammatory molecules in mouse microglial cells. Mouse BV-2 microglial cells were treated with different concentrations of NaPB for 6 h followed by stimulation with 1 µM MPP+ under serum-free condition. After 5 h of stimulation, the mRNA expression of iNOS, IL-1β and TNF-α was monitored by semi-quantitative RT-PCR (A) and quantitative real-time PCR (C, iNOS; D, IL-1β; E, TNF-α). After 24 h of stimulation, the protein expression of iNOS was monitored by Western blot (B). Results are mean + SD of three different experiments. ap<0.001 vs control; bp<0.001 vs MPP+. (TIF) [file pone.0038113.s002.tif]

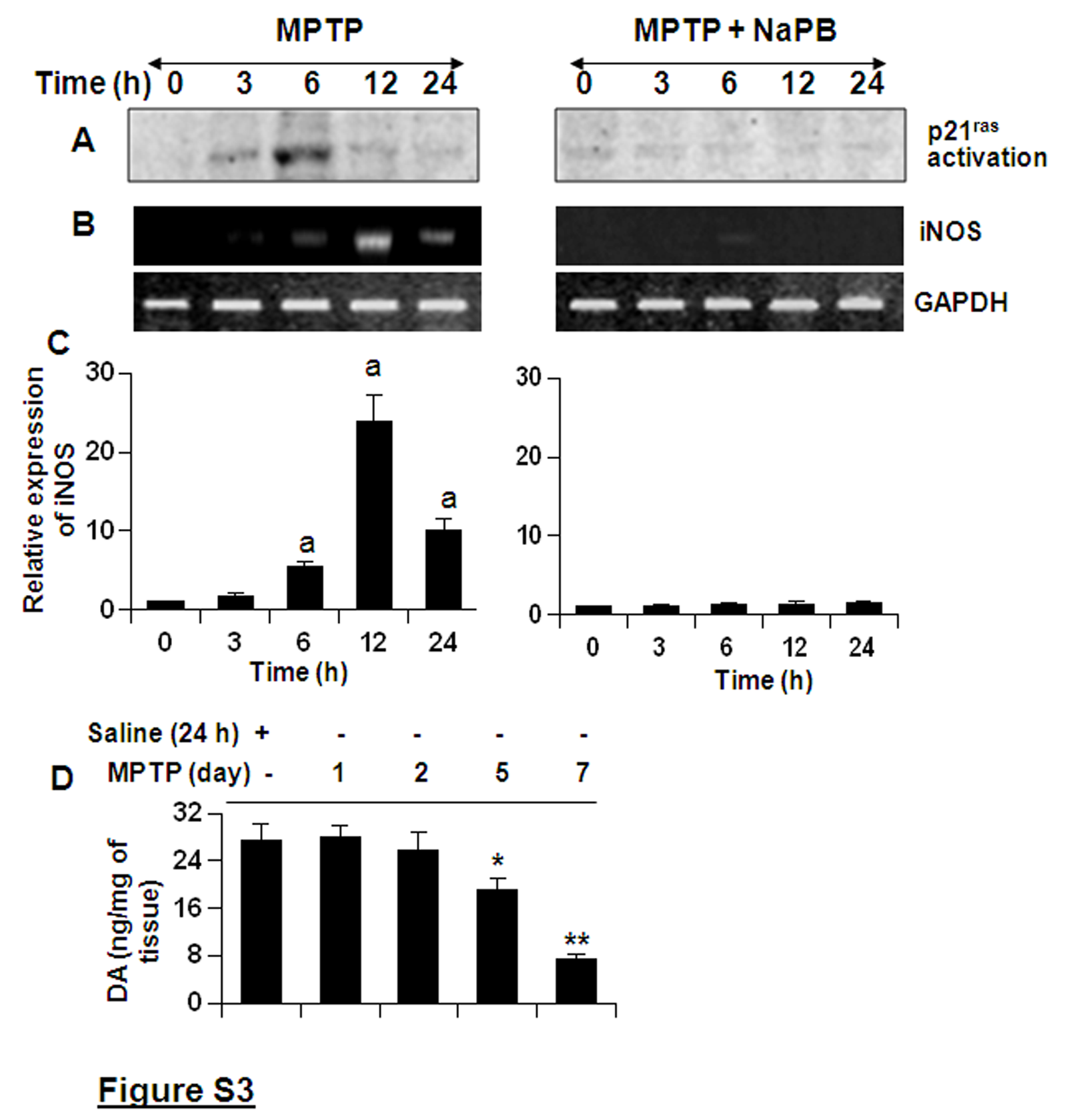

Supplement: Figure S3 — Time course of nigral activation of p21ras and expression of iNOS and striatal loss of dopamine in MPTP-intoxicated mice. A) Mice were treated with NaPB (200 mg/kg body wt/d) via gavage from 1 d prior to MPTP injection. At different h of MPTP insult, nigral punches from two mice were pooled together and analyzed for the activation of p21ras (A) and the expression of iNOS mRNA (B, RT-PCR; C, real-time PCR). Experiments were repeated three times each time using two animals in each group. ap<0.001 vs control (0h)-iNOS. D) At different days of MPTP insult, dopamine was quantified in striata. Data are means + SEM of six mice per group. *p<0.05 vs control; **p<0.001 vs control. (TIF) [file pone.0038113.s003.tif]

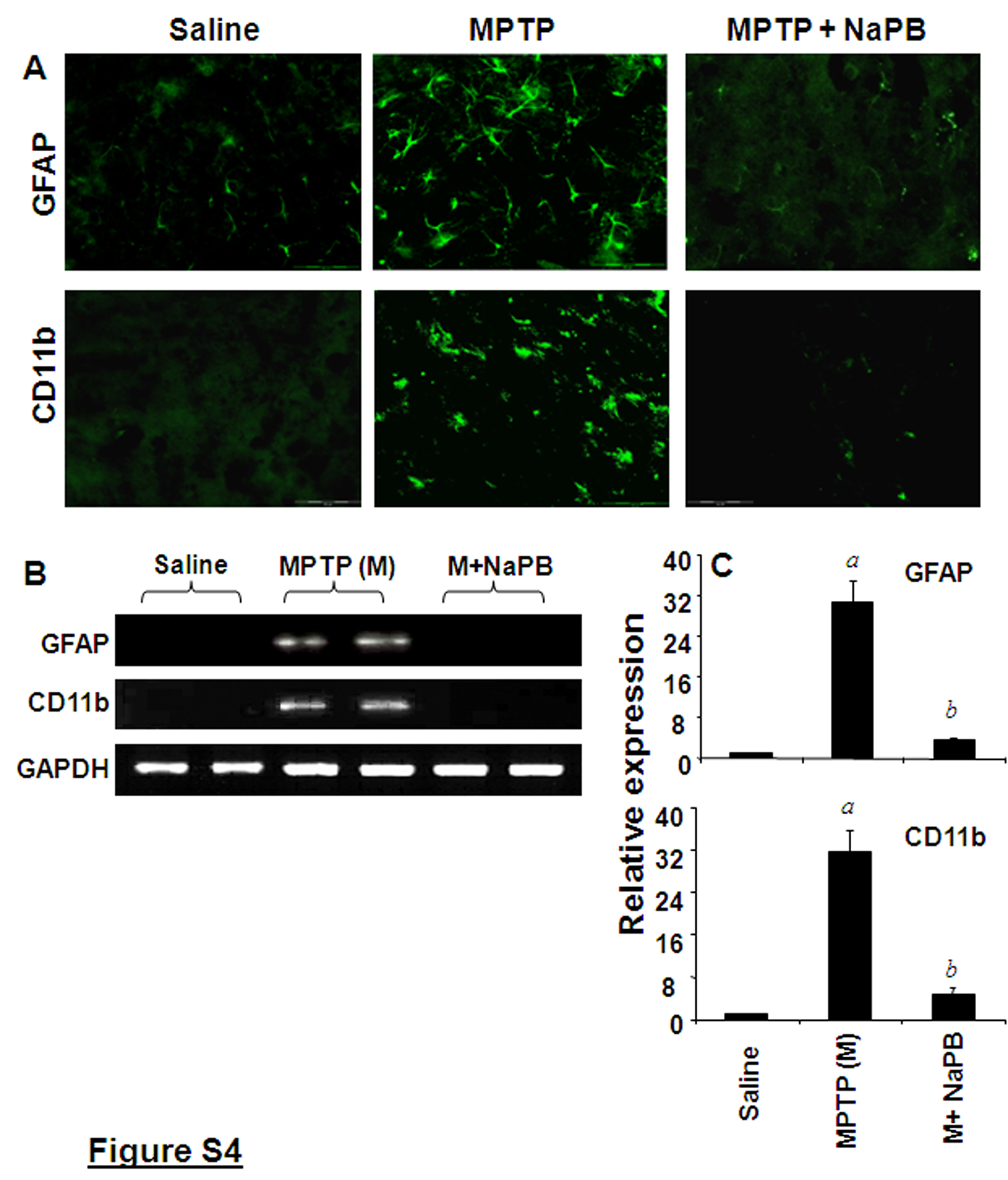

Supplement: Figure S4 — Increased expression of CD11b and GFAP in ventral midbrain of MPTP-intoxicated mice is NaPB-sensitive. A) Mice were treated with NaPB (200 mg/kg body wt/d) from 3 h after the last injection of MPTP. Twenty-four h after the last injection of MPTP, ventral midbrain sections were immunostained for GFAP and CD11b. Results represent analyses of four nigral sections (two images per slide) from each of four mice. The mRNA expression of GFAP and CD11b was analyzed by semi-quantitative RT-PCR (B) and quantitative real-time PCR (C). Data are means + SEM of five mice per group. ap<0.0001 vs saline group; bp<0.0001 vs the MPTP group. (TIF) [file pone.0038113.s004.tif]

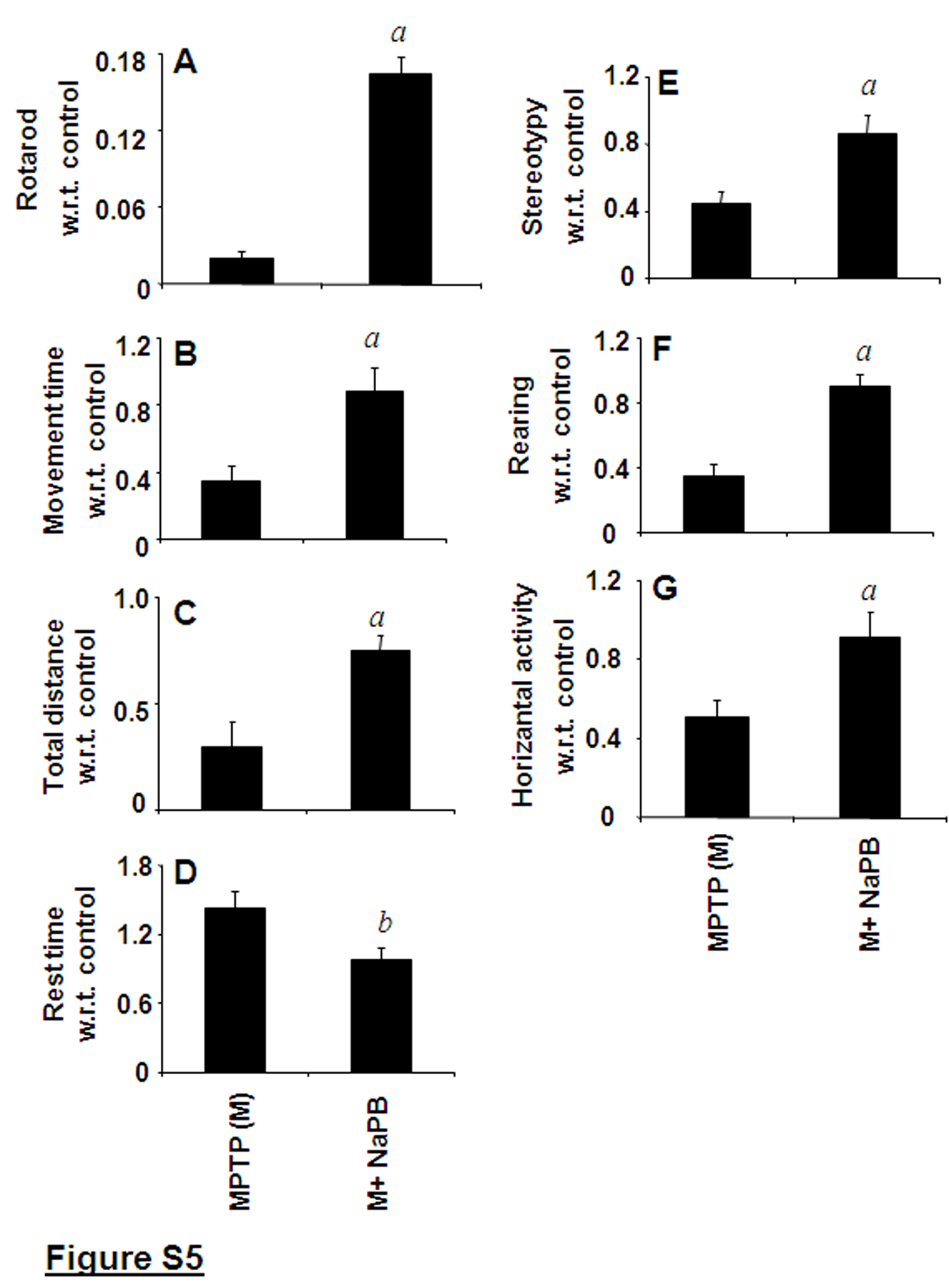

Supplement: Figure S5 — NaPB improves motor functions in MPTP-intoxicated mice. Mice receiving NaPB (200 mg/kg body wt/day) from 3 h after the last injection of MPTP were tested for motor functions (A, rotorod; B, movement time; C, total distance; D, rest time; E, stereotypy counts; F, rearing; G, horizontal activity) 7 d after the last injection of MPTP. Data are means + SEM of eight mice per group.ap<0.001 vs MPTP; bp<0.05 vs MPTP. (TIF) [file pone.0038113.s005.tif]
